# Modulatory Effects on Laminar Neural Activity Induced by Near-Infrared Light Stimulation with a Continuous Waveform to the Mouse Inferior Colliculus In Vivo

**DOI:** 10.1523/ENEURO.0521-23.2024

**Published:** 2024-05-07

**Authors:** Hiromu Sato, Futoshi Sugimoto, Ryo Furukawa, Takashi Tateno

**Affiliations:** ^1^Bioengineering and Bioinformatics, Graduate School of Information Science and Technology, Hokkaido University, Sapporo 060-0814, Japan; ^2^Division of Bioengineering and Bioinformatics, Faculty of Information Science and Technology, Hokkaido University, Sapporo 060-0814, Japan

**Keywords:** brain stimulation, near-infrared light, neural modulation, Inferior colliculus, photothermal effects

## Abstract

Infrared neural stimulation (INS) is a promising area of interest for the clinical application of a neuromodulation method. This is in part because of its low invasiveness, whereby INS modulates the activity of the neural tissue mainly through temperature changes. Additionally, INS may provide localized brain stimulation with less tissue damage. The inferior colliculus (IC) is a crucial auditory relay nucleus and a potential target for clinical application of INS to treat auditory diseases and develop artificial hearing devices. Here, using continuous INS with low to high-power density, we demonstrate the laminar modulation of neural activity in the mouse IC in the presence and absence of sound. We investigated stimulation parameters of INS to effectively modulate the neural activity in a facilitatory or inhibitory manner. A mathematical model of INS-driven brain tissue was first simulated, temperature distributions were numerically estimated, and stimulus parameters were selected from the simulation results. Subsequently, INS was administered to the IC of anesthetized mice, and the modulation effect on the neural activity was measured using an electrophysiological approach. We found that the modulatory effect of INS on the spontaneous neural activity was bidirectional between facilitatory and inhibitory effects. The modulatory effect on sound-evoked responses produced only an inhibitory effect to all examined stimulus intensities. Thus, this study provides important physiological evidence on the response properties of IC neurons to INS. Overall, INS can be used for the development of new therapies for neurological disorders and functional support devices for auditory central processing.

## Significance Statement

Using continuous infrared neural stimulation (INS) of low to high-power density, we sought to examine the laminar modulation of the neural activity in the mouse inferior colliculus (IC) in the presence and absence of sound. We found that the modulatory effect of INS on the spontaneous neural activity was bidirectional between facilitatory and inhibitory effects. Additionally, the modulatory effect on sound-evoked responses produced only an inhibitory effect at all examined stimulus intensities. Thus, this study provides important physiological evidence on the response properties of IC neurons to INS. Moreover, INS can be used for the development of new therapies for neurological disorders and functional support devices for auditory central processing.

## Introduction

Optical infrared neural stimulation (INS) is a promising area of interest for clinical application of a neural modulation method. To modulate the activity of neurons or neural tissue in an illumination spot of infrared light wavelength, INS uses a temporal pattern of low-intensity light to ensure the tissue is not damaged. Previous studies have shown that INS activates peripheral nerves (Wells et al., 2007a), peripheral sensory systems (Izzo et al., 2006), and cardiac tissue (Jenkins et al., 2010). In the central nervous system (CNS), several studies have reported INS-induced responses and activity modulation of the neural tissue in animal models, including rat thalamocortical slices in vitro (Cayce et al., 2010), rodent somatosensory cortex in vivo (Cayce et al., 2011), and nonhuman primate visual cortex in vivo (Cayce et al., 2014). Furthermore, because of its high spatial precision, studies of focal INS have recently been applied to mapping the brain connectome (Xu et al., 2019). However, little is known about the optical stimulation conditions of INS and its underlying mechanisms in specific brain areas, including the auditory CNS. Currently, the main mechanism for INS-induced cell excitation and activity modulation is believed to be the transduction of electromagnetic energy to thermal heat (Wells et al., 2005; Chernov and Roe, 2014), possibly owing to changes in ion channel activity (Albert et al., 2012) and heat-induced transmembrane capacitive charges (Shapiro et al., 2012).

The inferior colliculus (IC) is a critical integration center of the auditory pathway in mammals. All ascending outputs projecting from the lower auditory brainstem and a large descending projection from the auditory cortex converge in the IC (Adams, 1979; Glendenning and Masterton, 1983; Oliver, 1984, 1987; Winer et al., 1998). The IC is composed of core (central nucleus) and shell (dorsal and lateral nuclei) subdivisions based on distinct dendritic morphologies and axonal trajectories (Oliver and Morest, 1984). The IC core primarily receives ascending brainstem inputs (Cant and Benson, 2006), while physiological and behavioral studies have shown that the IC shell receives both ascending inputs (Loftus et al., 2008) and descending auditory cortex and IC core inputs (Winer et al., 1998, 2005). In response to the spectral and temporal content of sound, IC neurons show frequency-selective responses. Therefore, IC neurons can perform computations critical for sound localization and the identification of vocalization and other communication sounds (Winer et al., 2005; Felix et al., 2018). However, no previous studies have shown how INS can modulate the spontaneous activity and sound-evoked responses of IC neurons.

In this study, to modulate the spontaneous neural activity and sound-driven responses in the IC, we chose a near-infrared wavelength (*λ *= 1,940 nm) with higher absorption in the brain tissue and continuous waveforms of light irradiation for efficient heat generation in deeper brain areas. Few studies have used continuous-wave (CW) INS for neural modulation (Xia and Nyberg, 2019; Horvath et al., 2020), although repetitive short and pulsed patterns are widely applied (Cayce et al., 2011; Chernov and Roe, 2014; Kaszas et al., 2021; Pan et al., 2023). Continuous waveforms of laser irradiation at a lower-power density but over a relatively longer period (≥10 s) are expected to diffuse the heat from the brain surface and enable deeper penetration into the brain. This should result in safer and more effective modulation of the neural activity in a wider brain area. Thus, we expect this to be a more appropriate method for deeper brain stimulation. First, to determine a set of appropriate INS parameters, we numerically calculated thermal spatial distributions in a brain tissue model combining an INS thermal effect. To do this, we used a multiphysics software to simulate combined physical phenomena, that is, the thermal effect of INS, heat transfer from the tissue, and heat diffusion process. Second, on the basis of the estimated stimulation parameters, INS was applied to the IC of anesthetized mice. Neural responses were then recorded through a multichannel linear probe to investigate the lamina dependency of INS effects. Third, the multiunit activity (MUA) during INS was obtained from the neural responses, and changes in the firing rate were examined in accordance with stimulation intensity and lamina depth from the brain surface. Our results show that INS modulation of the spontaneous firing rate is depth-dependent and bidirectional with stimulation intensity. Altogether, our study provides physiological evidence from an animal model demonstrating the feasibility of INS for the clinical treatment of neural disease in humans and possible compensation of future auditory neural dysfunction.

## Materials and Methods

### Near-infrared light stimulation setup

**Figure 1.**
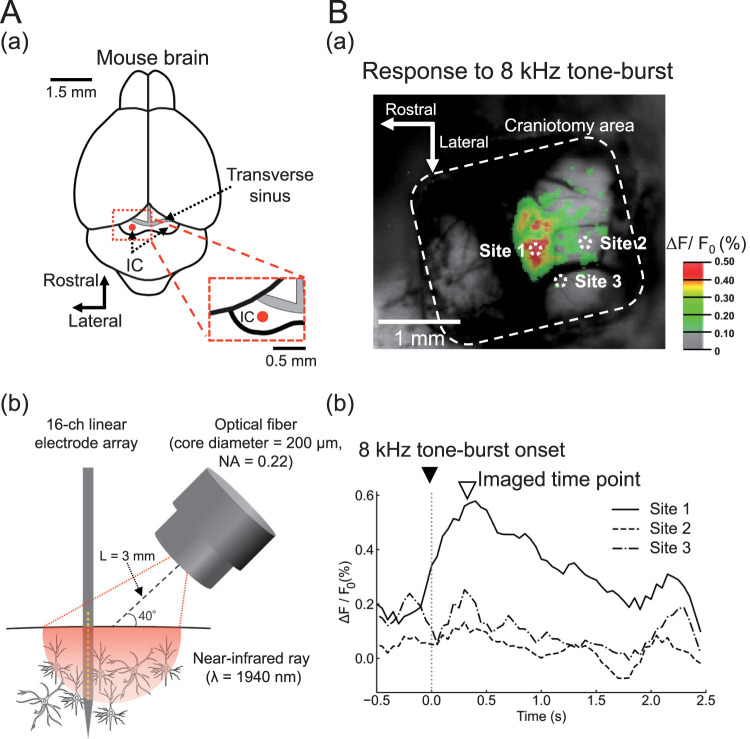
Near-infrared light stimulation to the mouse IC. ***A***, Schematic of a whole mouse brain and the location of the left IC in ***a***. Top view of the recording site (red circle) in the mouse left IC is illustrated in ***a***. The inset is the expanded view around the left IC. Schematic of an optical fiber for stimulation of near-infrared light (wavelength, *λ* = 1,940 nm) and a 16-channel (ch) linear silicon probe for recording of the neural activity from the IC in ***b***. The edge of an optical fiber was placed 3 mm from the brain surface (i.e., *L *= 3 mm) at a tilt angle of 40° with respect to the brain surface in ***b***. The core diameter of the optical fiber was 200 μm, and the NA was 0.22. ***B***, Flavoprotein autofluorescence imaging to locate sound-driven responses in the mouse IC in ***a***. In response to 8 kHz tone burst stimuli, time courses of flavoprotein imaging in several positions (sites 1–3) from the activated area (site 1) and surrounding areas (sites 2 and 3) in ***a*** are illustrated in ***b***. The timings of sound onset and imaged time points are respectively represented by black (▾) and white (∇) inverted triangles. See Extended Data Figure 1-1 for more details.

Near-infrared light stimulation was carried out using a semiconductor (gallium arsenide [GaAs]/aluminum GaAs [AlGaAs]-based laser (wavelength, *λ* = 1,940 nm) as a light source (LU1940D012 U10AA, s/n 98457; Lumics). Light was delivered to the mouse IC (Fig. 1*Aa*) via a 200-μm-diameter multimode optical fiber with a numerical aperture (NA) of 0.22 (Lu_LWL_200/280; Lumics) and a maximum light source output of 1.2 W. Using a micromanipulator, the optical fiber was positioned at ∼3 mm away from the brain surface above the mouse IC (Fig. 1*Ab*). The laser output was calibrated before each experiment using a power meter (PM101A; Thorlabs) with a thermal power head detector (S405C; Thorlabs). The output of the laser module was controlled by a function generator (WF1947; NF), which fed a trigger signal for generating laser output. The function generator was also synchronized by the trigger signal to a recording system of the neural activity.

10.1523/ENEURO.0521-23.2024.f1-1 Figure 1-1 **Flavoprotein autofluorescence imaging.** (A) Flavoprotein autofluorescence imaging to locate sound-driven responses in the mouse inferior colliculus in (a). In response to a 4 kHz tone burst stimulus, time courses of flavoprotein imaging in several positions (sites 1 to 3) of activated areas in (a) are illustrated in (b). (B) Similarly, in response to a 16 kHz tone burst stimulus. Timings of sound onset and imaged time points are represented by black (▾) and white (▽) inverted triangles, respectively. Download Figure 1-1, TIF file.

### Stimulation pattern and intensity of infrared light

Continuous (sustained) waveforms for laser irradiation were used, rather than repetitive short-pulsed waveforms, as have often been used in previous INS studies (Wells et al., 2005; Izzo et al., 2008; Cayce et al., 2011). Pulsed wave lasers repeatedly deliver heat to the brain surface over a very short period of time. This prevents rapid diffusion of the heat to the surrounding area and is expected to produce a temperature increase in only a small volume near the surface. In contrast, when using continuous waveforms of laser irradiation at a low-power density to the brain surface over a longer period of time, the heat is expected to diffuse and penetrate deeper, resulting in better modulation of the neural activity in wider and deeper brain areas. This should be a more appropriate method for deep brain stimulation, which is the anticipated outcome of this study. Each stimulation pattern was structured as a continuous waveform of infrared light. The stimulus duration was 30 s, and the interstimulus interval was 40 s, unless stated otherwise. The interstimulus interval was longer than numerically estimated and actually observed intervals for tissue temperature to drop to a prestimulus baseline; specifically, the approximate dropping period was 18 s even at a higher-power density (see Results). For one condition, a 70 s stimulus pattern, including the interval, was repeated at least five times for each parameter condition. Therefore, the only variable among the stimulation conditions was the radiant exposure intensity (power density) per stimulus. As the stimulus duration and interstimulus interval remained constant, the stimulation condition was changed by altering the laser input voltage within a range of output power, specifically from 15, 20, to 70 mW in 10 mW steps. The power intensity applied during stimulation was expressed as the power deposited at the brain surface. To calculate the effective area exposed to infrared light, we considered multiple parameters including the fiber core diameter, its NA, the angle between the mouse brain surface and optical axis, the width of the cranial window, and different refractive indexes (i.e., immersion solution and brain tissue). After calculation (see below, a computational model estimation to select stimulation parameters), the brain surface exposed to infrared light was found to be an ellipse with a large axis of 0.330 cm and a small axis of 0.204 cm, representing a total area of ∼0.214 cm^2^. Powers of 15, 20, and 70 mW correspond to 70.1, 93.5, and 327 mW/cm^2^ power densities, respectively, at a saline layer on the brain surface. In our calculation of power density, the absorption of immersion solution on the brain surface was also considered. In each session, the order of stimulation intensities was randomized, although INS at the highest intensity (70 mW) was always presented in the last trial series to avoid severe damage to the brain tissue.

### Surgical procedure

All animal procedures were performed in accordance with Hokkaido University Animal Care Committee's regulations and in accordance with the NIH Guidelines for the Care and Use of Laboratory Animals. A total of 13 (five males and eight females) C57BL/6J mice (8–13 weeks old; Japan SLC) were used. Mice were kept under a 12:12 h light–dark cycle [zeitgeber time (ZT)0 = 07:00] in a temperature-controlled room at 24 ± 1°C. Mice were fed standard food pellets and tap water, which was available *ad libitum*. Urethane (1.5 g/kg; Wako) was used to anesthetize the mice. After exposing the skull, a miniature screw was imprinted into the skull at 2–3 mm caudal to the lambda and used as a reference electrode during the recording of the neural activity. A craniotomy and durotomy were performed to expose the IC (2–3 mm caudal to the lambda and 2 mm lateral to the midline). Saline was dripped onto the brain surface to prevent dryness. The body temperature was maintained at 37°C using a heating pad. To detect auditory responses, we used a flavoprotein autofluorescence. Our experimental method for optical imaging is based on that proposed by Shibuki et al. (2009). All animal experiments were performed in a dark soundproofed room with all lights turned off. However, uneven and wet surfaces could lead to variations in blue light reflection and scattering, potentially causing distortions in imaging results and contributing to image artifacts against actual neural responses. Briefly, a complementary metal–oxide–semiconductor (CMOS) camera system (MiCAM02; Brainvision) was mounted on a tandem-lens upright microscope (THT; Brainvision). Excitation light was provided by a 465 nm blue light-emitting diode (LEX2-B; Brainvision) through a blue bandpass filter (466 ± 20 nm). Using the CMOS camera system with a dichroic mirror and green bandpass filter (525 ± 22.5 nm), endogenous flavoprotein green fluorescence (500–550 nm) was detected. Images (188 × 160 pixels; 30 µm/pixel) of fluorescence signal in the mouse IC were recorded at 20 frames/s before and after tone burst sound stimulation to examine the frequency selectivity (Fig. 1*Ba*). Trials were repeated 10 times at 8–10 s random intervals, and averaged images were displayed using an acquisition and analysis software (BV_Ana; Brainvision). Spatial averaging of 5 × 5 pixels and temporal averaging of five consecutive frames were used for further analysis (Fig. 1*Ba*,*b*). Fluorescence intensities in the obtained images were normalized pixel by pixel with respect to the reference image (*F*_0_), obtained 500 ms before acoustic stimuli administration. The total imaging duration per trial was 3.0 s. To show changes in fluorescence (Δ*F*), we presented Δ*F*/*F*_0_ as a percentage (Fig. 1*Ba*,*b*). After verifying the auditory response area, the linear electrode array was inserted into the IC core (see below, Multichannel electrophysiology). The preparatory procedure from the initial anesthesia until the opening of the skull and insertion of the electrode array lasted ∼50–150min.

### Multichannel electrophysiology

Electrophysiological experiments were performed in a *shielded* soundproof room. Multichannel electrophysiological recordings were conducted with a single-shank 16–channel electrode array at 50 µm spacing (0.98–2.03 MΩ at 1 kHz; A1×16-5 mm-50-177-A16; NeuroNexus Technologies). The single-shank electrode array was inserted into a site in the core region of the IC. MUAs and local field potentials (LFPs) were recorded using an amplification gain of 250 with bandpass filters in a frequency range of 300–8,000 and 0.1–300 Hz, respectively, in combination with a 50 Hz notch filter to remove the power line noise (OmniPlex; Plexon). Spikes and LFPs were sampled at rates of 40 and 1 kHz, respectively. To sort MUAs, we used filtered waveforms obtained from originally recorded data. Multiunit spikes were detected by threshold processing with 4.0 × sigma (*σ*; negative crossing of the threshold), where *σ* is the standard deviation of filtered data.

### A computational model estimation to select stimulation parameters

To explore effective stimulation parameters for INS, we constructed a computational model based on a physical structure of the brain tissue and infrared light irradiation. A bioheat equation provides a standard description of heat transfer within the biological tissue. It accounts for thermal conduction and heat storage in a living tissue, following Pennes' approximation (Pennes, 1948; Elwassif et al., 2006):

(1) $$\rho C_{p}\frac{dT}{dt}-\nabla \cdot (k\nabla T)=Q_{\mathrm{perf}}+Q_{e}+Q_{\mathrm{met}},$$

where *ρ* is the density of a medium (brain tissue), *T* is the temperature of the medium, *C_p_* is the heat capacity, and *k* is the thermal conductivity. The three transfer terms (*Q*_perf_, *Q_e_*, and *Q*_met_) on the right-hand side of Equation (1) represent blood perfusion cooling, electromagnetic (laser) heating, and metabolic heating terms, respectively. Convective cooling of blood perfusion was modeled by the transfer term *Q*_perf_:

(2) $$Q_{\mathrm{perf}}=\rho_{b}C_{b}\omega_{b}(T_{b}-T),$$

where *ρ_b_* is the blood density, *C_b_* is the blood heat capacity, *ω_b_* is the blood perfusion rate, and *T_b_* is the blood temperature. The blood perfusion rate, *ω_b_*, controls the amount of cooling, and the rate may depend on space, time, temperature, or health status of the tissue. Heat generation from metabolism can be accounted for in the *Q*_met_ term. In addition, the heat source of the Gaussian laser beam, *Q_e_*, was modeled by the following equation (Peixoto et al., 2020):

(3) $$Q_{e}=\;\frac{Q_{0}\mu_{a}}{2\pi \sigma_{x}\sigma_{y}}\exp\left[-\left(\frac{{\left(x-x_{0}\right)}^{2}}{2\sigma_{x}^{2}}\right)\;-\left(\frac{{\left(y-y_{0}\right)}^{2}}{2\sigma_{y}^{2}}\right)\right]\cdot g_{\mathrm{loss}}(d)\cdot F_{T}(d),$$

where *Q*_0_ is the laser light output, *μ_a_* is the absorption coefficient of the tissue, and *σ_x_* and *σ_y_* are the respective beam radii. In Equation (3), considering both scattering and absorption effects (Peixoto et al., 2020), the transmission fraction, *F_T_* (as a function of distance *d* from the optic fiber), was described as follows:

(4) $$F_{T}(d)=\frac{b}{a\cdot \sinh(a\mu_{s}d)+b\cdot \cosh(b\mu_{a}d)},$$

where *a* and *b* are given by the following:

(5a) $$a=1+\frac{\mu_{a}}{\mu_{s}},$$

(5b) $$b=\sqrt{a^{2}-1},$$

and *μ_s_* is a scattering coefficient. In addition, geometric loss is the fractional decrease in intensity from the tissue surface (at *z *= 0) owing to the conical geometry of the emitted light at a given distance (*d*) in the absence of tissue scattering and absorption. Considering the conservation of energy, geometric loss, *g*_loss_, can be calculated to a given distance *d*, in the tissue as follows (Aravanis et al., 2007):

(6) $$g_{\mathrm{loss}}(d)=\frac{\rho_{0}^{2}}{{\left(d+\rho_{0}\right)}^{2}},$$

with

(7) $$\rho_{0}=r\sqrt{{\left(\frac{n_{\mathrm{tis}}}{NA_{\mathrm{fib}}}\right)}^{2}-1},$$

in which *r* is the fiber core radius, *n*_tis_ is the refractive index of the tissue (around a value of 1.36–1.5), and NA_fib_ is the NA (0.22) of the optical fiber. All parameters used are listed in Table 1. To simulate the model, we used the COMSOL Multiphysics software (Version 6.0; COMSOL). INS intensities of light were varied in COMSOL Multiphysics to numerically calculate the temperature distribution in the brain tissue. All parameter values used in the computational model are summarized in Table 1.

**Table 1.** Computational model parameters

Symbol	Parameter in the brain tissue	Value	Reference
ρ	Density of the tissue	1,040 kg/m^3^	Elwassif et al. (2006)
$C_{p}$	Specific heat of the tissue	3,650 J/kg°C	Elwassif et al. (2006)
k	Thermal conductivity of the tissue	0.527 W/m°C	Elwassif et al. (2006)
$Q_{\mathrm{met}}\;$	Metabolic heat	13,698 W/m^3^	Elwassif et al. (2006)
$Q_{0}$	Generated heat by infrared ray	15–70 mW	(same as the experimental condition)
$R_{c}$	Reflection coefficient	0	Peixoto et al. (2020)
$\mu_{a}$	Absorption coefficient	97.68 cm^−1^	Jacques (2013)
$\mu_{s}$	Scattering coefficient	11.3 cm^−1^	Jacques (2013)
$\sigma_{x}\;$	Standard deviations in the *x*-axis	1.65 mm	(same as the experimental condition)
$\sigma_{y}$	Standard deviations in the *y*-axis	1.02 mm	(same as the experimental condition)
$x_{0},y_{0}$	Center of laser spot	(0, 0) mm	(same as the experimental condition)
$\rho_{b}$	Density of the blood	1,057 kg/m^3^	Elwassif et al. (2006)
$C_{b}$	Specific heat of the blood	3,600 J/kg°C	Elwassif et al. (2006)
$\omega_{b}$	Blood perfusion	0.012 s^−1^	Elwassif et al. (2006)
$T_{b}$	Blood temperature	36.7°C	Elwassif et al. (2006)
*n*_tis_	Refractive index of the brain tissue	1.36	Vo-Dinh (2014) and Peixoto et al. (2020)
$NA_{\mathrm{fib}}$	NA of the optical fiber	0.22	(same as the experimental condition)

### Code accessibility

All computational models simulated in this study were implemented using COMSOL Multiphysics. To facilitate transparency and reproducibility, the model setting files (file extensions, “mph”) for the numerical simulations are available for download alongside this manuscript. The complete set of code files, including additional documentation, has been compressed into an archive file.

10.1523/ENEURO.0521-23.2024.d1 Extended Data Download Extended Data, ZIP file.

### Sound stimulation

To locate the IC during the flavoprotein autofluorescence imaging, we generated the click sound stimuli using a digital-to-analog converter (NI USB-6341; National Instruments) at a 500 kHz sampling rate, amplified with a stereo amplifier (SA1; Tucker-Davis Technologies). Click pulse trains [sound intensity, 60 dB *sound pressure level (*SPL); duration, 100 µs; monophasic pulse] were presented 20 times at random 3.9–4.1 s intervals when recording LFPs in the IC. Similarly, pure tone burst sounds (frequency, 4, 8, and 16 kHz; sound intensity, 60, 70, or 80 dB SPL; duration, 100 ms) were used to measure the characteristics of sound responses. Here, we used the linearly increasing onset and decreasing offset of stimulus envelops, which were respectively set at 10% of the total duration of each stimulus (Tsytsarev and Tanaka, 2002). Sound stimuli were presented via a speaker (MF1; Tucker-Davis Technologies). Prior to starting each experiment, stimuli were calibrated with a sound level meter (Type 2636; Brüel & Kjaer) and a 0.25 in microphone (Type 4939-L-002; Brüel & Kjaer). To characterize IC lamina properties, we applied a standard current source density analysis (Nicholson and Freeman, 1975) for each sound-induced response. The noise level of the soundproof room was <24 dB SPL without acoustic stimulation.

### Drug application on the brain surface

To examine the involvement of temperature-sensitive ion channels in the neural modulatory effect of INS, we applied a 20 µM ruthenium red (RR), an antagonist of transient receptor potential (TRP)V1–4 and TRPA1 channels, to the exposed IC surface. After a sufficient infiltration period (∼50 min), the same light stimulation and recordings of evoked responses were performed.

Furthermore, to examine whether spike signals originated from the actual neuronal activity, we applied a 20 µM tetrodotoxin (TTX), which is an antagonist of voltage-gated sodium channels, to the brain surface after a series of experimental sessions were completed. Then, extracellular signals possibly including spontaneous neuronal activities were recorded, again after an infiltration period (∼50 min). TTX and RR applications were administered to different individual animals to avoid the drug effects of each other (Osanai et al, 2018).

### Data analysis

Raster plots represent firing timings (MUAs) of neurons recorded in individual electrodes. To examine firing rate changes during INS, peristimulus time histograms (PSTHs) show the total firing amount of neurons recorded from electrodes during constant time bins (1.0 s). In addition, to quantitatively evaluate changes in mean firing rates before and during INS, we calculated the two mean PSTH values, *F*_pre_ and *F*_stim_. *F*_pre_ is the mean PSTH value obtained during the 20 s period before the INS onset, whereas *F*_stim_ is the mean PSTH value obtained during a 20 s period within a 30 s stimulation period (excluding the 5 s periods after onset and before offset). The ratio (*F*_stim_/*F*_pre_) of the two values (i.e., *F*_pre_ and *F*_stim_) was then used to compare the results obtained with different INS powers. A ratio >1.0 represents an increase of the firing rate during the INS period compared with the prestimulation period, while <1.0 represents a decrease. Similarly, to examine whether the poststimulation neuronal activity returned to the baseline state before INS, we calculated the ratio (*F*_post_/*F*_pre_), where *F*_post_ is the mean PSTH value obtained during the 20 s period immediately after INS. In addition, *F*_post_5-20s_ is defined as the mean PSTH value obtained during the period between 5 and 20 s after the offset of INS. That is, *F*_post_5-20s_ is a mean PSTH variable that excluded firing rates in the period during 5 s immediately after the stimulation offset.

Furthermore, to determine whether sound-evoked responses during simultaneous INS are modulated, the following analysis was performed. First, PSTH values (*f*_PSTH_) were calculated from the obtained MUAs in response to sound (duration and sound type; 0.1 ms click or 100 ms tone burst) and INS (duration, 30 s). Next, a standardization was performed by calculating the *Z* score by subtracting the average firing rate (*m*) from *f*_PSTH_ during the simultaneous stimulation period and dividing by its standard deviation (*σ*), that is, *Z* score was denoted by (*f*_PSTH_ − *m*)/*σ*. This normalization was performed in the time interval from −0.1 to 1.0 s of the measurement data, while the acoustic stimulus was presented at time 0 s. After standardization, the average *Z* score value was used to account for the type of sound stimulation with a 0.1 ms click and 100 ms tone burst sound (frequency, 4, 8, and 16  kHz). In this experiment, trials of sound click and tone bursts were presented 20 times at random 1.9–2.1 s intervals, when no INS was applied.

### Statistical analysis

All statistical analyses were performed using order statistics without assuming a specific distribution and employing nonparametric statistical methods. Characterization of firing properties modulated by INS in spontaneous activities of IC laminae was analyzed using a Kruskal–Wallis test with Steel's multiple-comparison test as a post hoc analysis. The modulatory effect of INS on neural sound-evoked responses was characterized by a Friedman test and Wilcoxon signed-rank test with Bonferroni’s correction as a post hoc analysis. Evaluation of changes in spontaneous firing properties during INS with RR administration was conducted with a Wilcoxon signed-rank test. A *p* value <0.05 was considered statistically significant. Python 3.9 and the statistical analysis software “EZR” (Easy R) were used for all statistical analyses (Kanda, 2013). The reported data are presented as mean ± standard error of the mean. Details of the statistical tests are summarized in Extended Data Table 1-1.

10.1523/ENEURO.0521-23.2024.t1-0 Table 1-0 Download Table 1-0, XLSX file.

10.1523/ENEURO.0521-23.2024.t1-1 Table 1-1 Download Table 1-1, XLSX file.

10.1523/ENEURO.0521-23.2024.t1-2 Table 1-2 Download Table 1-2, XLSX file.

## Results

### Simulation of thermal propagation in a brain tissue model by infrared light irradiation

**Figure 2.**
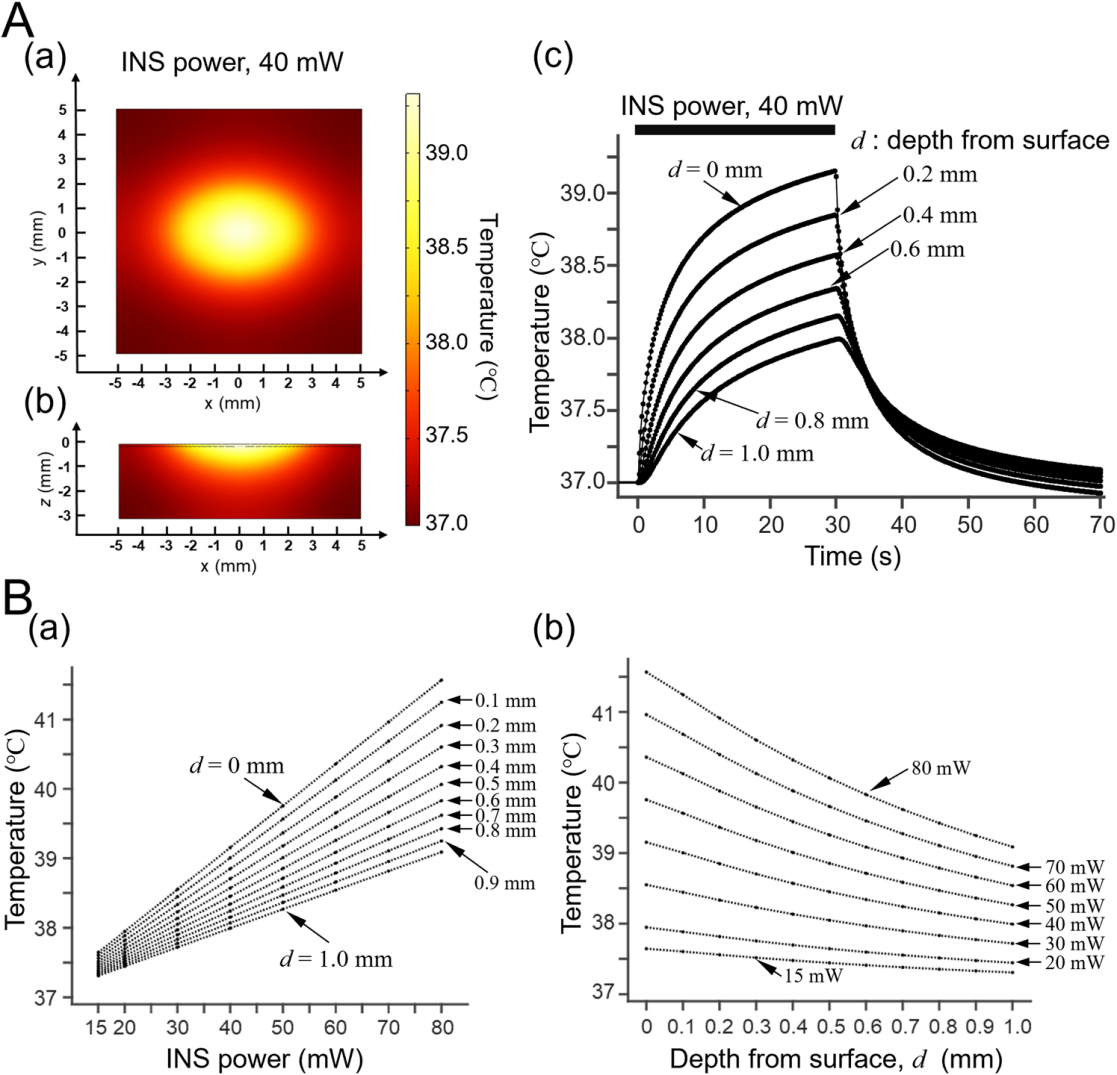
Computational simulation of temperature changes to INS in a brain tissue model. ***A***, Numerical simulation of temperature distributions in a brain tissue model projected to a *x*–*y* representation at *z *= 0 mm in ***a*** and *x*–*z* representation at *y *= 0 in ***b***, immediately after offset of INS (i.e., 30 s after onset). Six time courses at depths of 0, 0.2, 0.4, 0.6, 0.8, and 1.0 mm from the brain surface are illustrated in ***c***. Stimulation duration of near-infrared light stimulation is indicated by a black bar at the top (duration, 30 s); the power intensity was 40 mW. ***B***, Temperature changes 30 s after INS onset versus light irradiation power in ***a*** and depth from the surface (***d***) in ***b***. The data set for ***a*** and ***b*** was the same, although the graphs are different (i.e., the relationship against temperature). See Extended Data Figures 2-1–2-3 for more details.

To explore appropriate intensities of INS to modulate the neural activity, we first simulated a brain tissue model (rectangular space, 10 × 10 × 3 mm^3^; Extended Data Fig. 2-1) combining a near-infrared light source model [Eq. (3)]. Spatial temperature rises in the brain tissue model were numerically calculated in response to 30 s of INS with power intensities from 15 to 80 mW. Spatial distribution at the temperature peak at 30 s after the stimulation onset is shown in Figure 2*Aa*,*b* (power intensity, 40 mW). Under the center of the light source (*x *= 0; *y *= 0), we also analyzed the time course of temperature changes at several depths, ranging from the surface (*z *= 0 mm) to *z *= −1.0 mm in 0.2 mm steps (initial temperature, 37°C; Fig. 2*Ac*). These numerical results suggested that an irradiation power of 40 mW was enough to cause a temperature rise of 2°C at the brain surface [depth (*d*) = 0 mm; Fig. 2*Ba*,*b*]. In addition, an irradiation power of 50 mW caused a temperature rise of 2°C at a depth of 0.4 mm (*z *= −0.4 mm) from the surface, resulting in the possibility of modulating neural firing in layers 3/4 of the mouse brain tissue (Fig. 2*Ba*,*b*). Furthermore, to support the possibility, it is known that the modulation of neural firing and resultant changes in neural network function are evident for the temperature increase of at least 1°C during a short period (Kim et al., 2022); brain functions associated with animal behaviors are likely to be affected by a sustained 2°C increase (Picot et al., 2018; Peixoto et al., 2020). However, Horváth et al. recently reported that the modulation of neural firing can be induced in mouse hippocampal neurons when the local brain temperature is increased over 4°C (Horvath et al., 2020). Therefore, in this study, we selected power intensities ranging from 15 to 70 mW as a stimulation range. Additionally, it is noteworthy that, in several conditions of stimulation parameters close to the temporal patterns we used here, our simulation result suggests that repetitive short pulses (pulse width, 20 ms; repetition rate, 0.1, 0.3, 1.0, 3.0, and 10 Hz; stimulation duration, 30 s) need more light energy (or power) to provide the same heat (temperature increase Δ*T *= 1.5°C) at deeper layer (e.g., 800 μm from the brain surface) than the CW light stimulation (Extended Data Fig. 2-2). Furthermore, the numerical simulation of energy density distributions (unit, J/cm^2^) in a brain tissue model is illustrated in Extended Data Figure 2-3, which corresponds to Figure 2*Ba* demonstrated with temperature.

10.1523/ENEURO.0521-23.2024.f2-1 Figure 2-1 **Mesh structure of a three-dimensional brain tissue model** Three-dimensional (3D) structure for finite element method (FEM) modeling to numerically obtain temperature changes in the brain tissue model. For simplicity, an optical fiber is not drawn, but has a tilt angle of 40° to the brain surface. The 3D structure of the brain tissue model in (A) and projection to two-dimensional planes; *x-y* plane projection in (B) and *x-z* projection in (C). There is a saline layer on top, with a depth of 100 μm. Download Figure 2-1, TIF file.

10.1523/ENEURO.0521-23.2024.f2-2 Figure 2-2 **INS intensities of repetitive pulsed stimulation and continuous wave stimulation**. For repetitive pulsed stimulation, intensities need to provide the same heat (temperature increase Δ*T *= 1.5℃) at deeper layer (800 μm from the brain surface) were numerically calculated when the repetition rate was one of 0.1, 0.3, 1.0, 3.0, and 10  Hz. In the repetitive pulsed stimulation, the pulse width was 20  ms, and the stimulation duration was 30  s. In the continuous wave light stimulation (the rightmost point indicated by an arrow), similarly, the stimulation duration was 30  s and the layer was 800 μm from the brain surface. In the case, INS intensity was 30  mW. Download Figure 2-2, TIF file.

**Figure 3.**
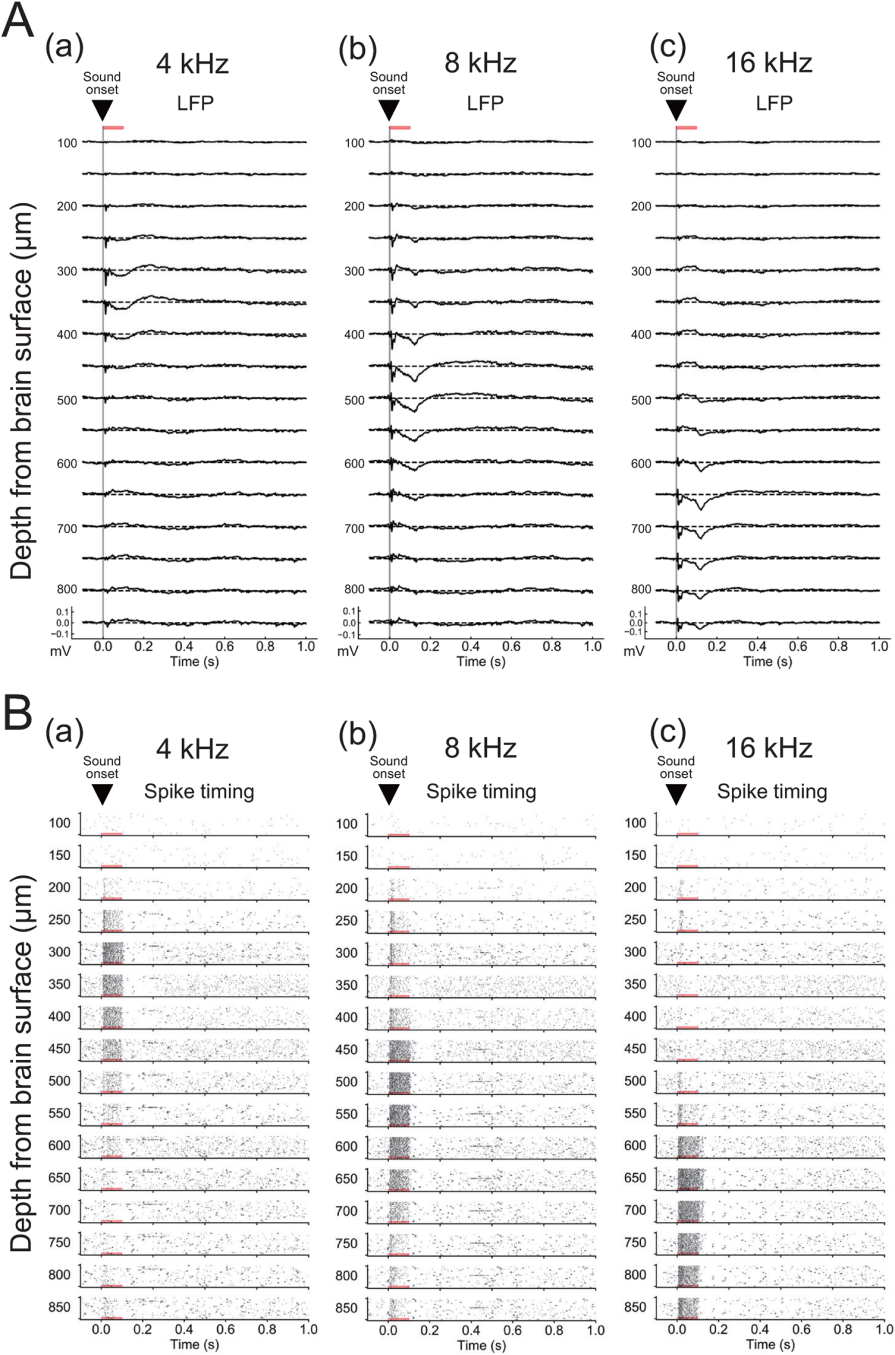
Sound-driven laminar responses in the mouse IC to tone bursts of different frequencies. ***A***, In response to sound stimulation (tone bursts of 4, 8, and 16 kHz with 60 dB SPL; duration, 100 ms), LFPs were recorded using a 16-channel electrode array in IC laminae (100–850 μm from the brain surface in 50 μm steps). The timings of the sound onset are represented by triangles. ***B***, Raster plots (spike timing) of MUA in response to sound stimulation (tone bursts of 4, 8, and 16 kHz with 60 dB SPL). Representations of 20 responses to tone bursts are shown in each raster plot, and the depth from the surface is indicated by numbers on the left (100–850 in μm). The timings of sound onset are also represented by inverted triangles. See Extended Data Figures 3-1–3-3 for more details.

10.1523/ENEURO.0521-23.2024.f2-3 Figure 2-3 **Light energy distribution in a brain with the unit of J/cm^2^**. Numerical simulation result of energy density (unit, J/cm^2^) as the function of the input powers (15, 20, 30, 40, 50, 60, and 70  mW) 30  s after INS onset, whose changes depended on the distance from the brain surface: 0, 0.3, 0.5, 0.8, and 1.0  mm. The INS stimulation duration was 30  s. Download Figure 2-3, TIF file.

### Properties of sound-driven neural responses in the mouse IC

Before inserting the electrode array into the IC, we conducted flavoprotein autofluorescence imaging to locate the IC areas responsive to sound stimulation (Fig. 1*B*; Extended Data Fig. 1-2). Average changes in fluorescence intensity in activated areas of the mouse IC in response to an 8 kHz tone burst sound in an anesthetized mouse are shown in Figure 1*Ba*. Onset sites of sound-evoked responses had a characteristic frequency preference. Specifically, areas responsive to a tone burst sound of a lower frequency (4 kHz) were located in rostral regions (Extended Data Fig. 1-1*A*), whereas those to a higher frequency (16 kHz) were found in caudal regions (Extended Data Fig. 1-1*B*). Additionally, the time course for fluorescence intensity changes at specific regions of the IC (site 1, dotted circle point in Fig. 1*Ba*) showed a peak latency of 0.39 ± 0.08 s from the sound onset of an 8 kHz tone burst stimulus (*n *= 4; Fig. 1*Bb*).

After examining the location of the IC area, a 16-channel linear electrode array substrate was inserted into the localized area where the flavoprotein imaging responses to the sound were most intensively detected (cf., site 1 in Fig. 1*Ba*). Spontaneous firings were always observed on extracellular recordings using the electrode array substrate. The firing rates of MUA were IC lamina-dependent: 73.1 ± 23.5 spikes/s in shallow laminae (*z *= −0.20 mm), 103.3 ± 21.2 spikes/s in middle laminae (*z *= −0.50 mm), and 162.9 ± 25.5 spikes/s in deeper laminae (*z *= −0.80 mm; *n *= 6)^aT^.

Furthermore, evoked LFP responses at brain depths ranging from 100 to 850 µm were recorded by two types of sound stimulation: (1) a tone burst sound at different frequencies (4, 8, and 16 kHz) and (2) a click sound. A tone burst sound at different frequencies evoked characteristic responses showing depth dependency (Fig. 3). That is, tone burst stimuli with a lower frequency (4 kHz) tended to evoke the activity in shallow laminae (≤450 μm; LFPs in Fig. 3*Aa*; MUAs in Fig. 3*Ba*), whereas those with a higher frequency (16 kHz) evoked the activity in deep laminae (≥600 μm; LFPs in Fig. 3*Ac*; MUAs in Fig. 3*Bc*). In addition, tone burst stimuli within a middle frequency (8 kHz) tended to evoke the activity of middle laminae (LFPs in Fig. 3*Ab*; MUAs in Fig. 3*Bb*). This result is consistent with previous studies (Stiebler and Ehret, 1985; Portfors et al., 2011). Furthermore, biphasic LFP responses with two negative-going peaks were observed according to onset and offset timings, particularly for tone burst stimuli with higher frequencies (8 and 16 kHz) in the middle (*z *= −300 to −450 μm; Fig. 3*Ab*,*c*) and deeper laminae (*z *= −450 to −600 μm; Fig. 3*Ab*,*c*). Altogether, we observed a similarity between the magnitude of LFP responses and higher MUA firing rates with respect to the frequency preference of tone burst sound; therefore the characteristic properties of LFP magnitude and MUA firing rate are consistent.

In contrast, a click sound evoked LFP responses in almost all channels except those in surface laminae (Extended Data Fig. 3-1). In shallow laminae (*z *= −200 μm from the surface), the fast negative-going peak had a latency of 24.2 ± 1.2 ms and amplitude of 0.134 ± 0.025 mV; in middle laminae (*z *= −500μm), the latency was 22.5 ± 0.8 ms, and the amplitude was 0.218 ± 0.019 mV; while in deeper laminae (*z *= −800 μm), the latency was 19.5 ± 1.8 ms, and the amplitude was 0.194 ± 0.016 mV. However, no significant differences were observed among laminae latencies and amplitudes (*n *= 6)^bT^. Because the click sound stimulus contains broadband frequency components, including 4–16 kHz frequencies, the short stimulus caused broad monophasic responses in shallow to deep IC laminae.

10.1523/ENEURO.0521-23.2024.f3-1 Figure 3-1 **Sound-driven laminar responses to a click sound in the mouse inferior colliculus.** (A) In response to click sound stimulation (60  dB SPL; and duration, 0.1  ms), local field potentials (LFPs) were recorded using a 16-channel electrode array in inferior colliculus laminae (100 to 850 μm from the brain surface in 50 μm steps). The timing of sound onset is represented by an inverted triangle. (B) Raster plots of multi-unit activity in response to click sound stimulation are shown, and correspond to the same experimental trials in (A). In each raster plot, 20 representations of responses to a click sound are shown; the depth from the surface is indicated by numbers (100 to 850 in μm) on the left-hand side. The timing of sound onset is represented by an inverted triangle. Download Figure 3-1, TIF file.

### Infrared light-driven bidirectional neural modulation in the mouse IC

**Figure 4.**
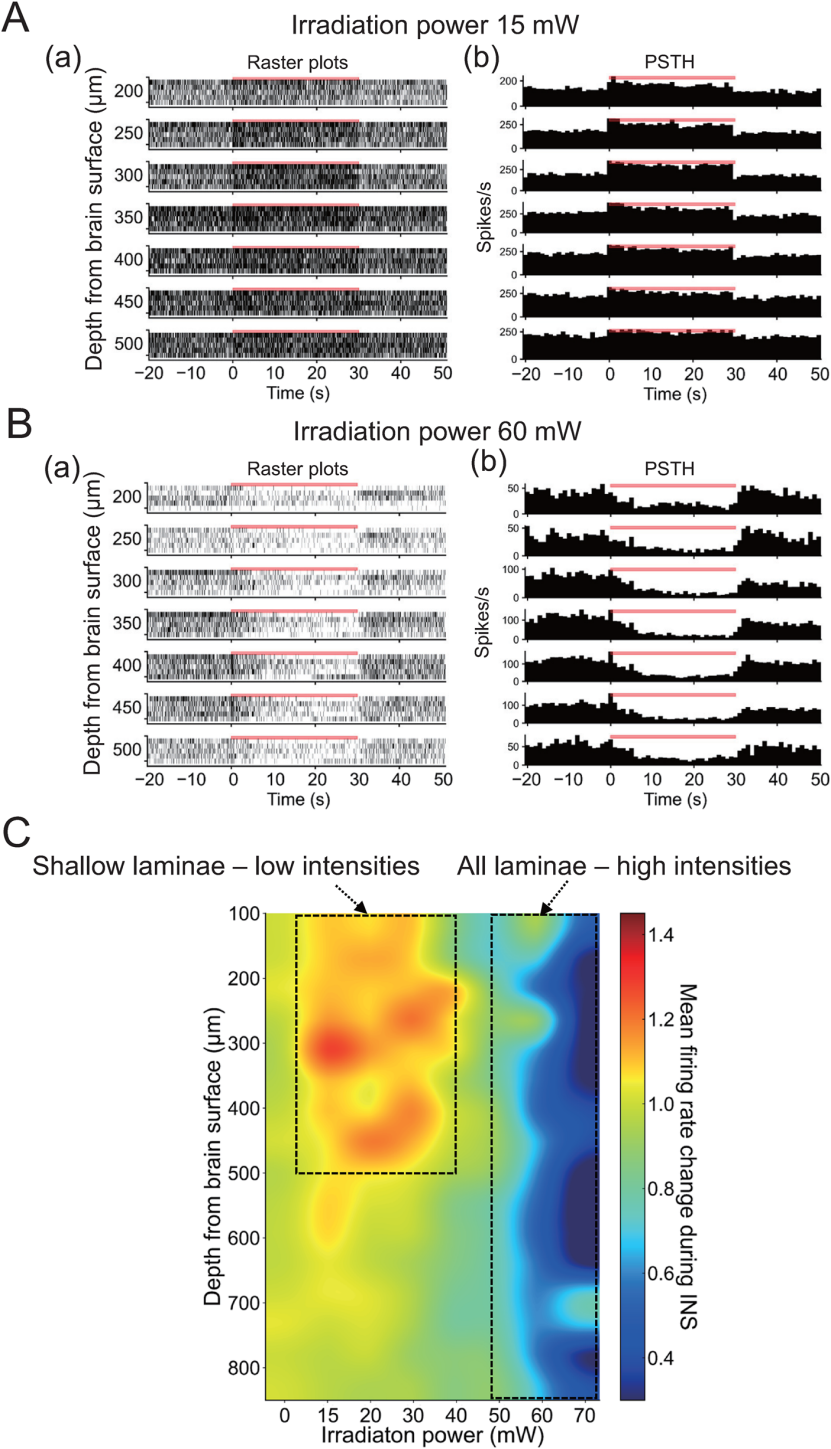
INS-induced modulation of the neural activity in the mouse IC. ***A***, In response to INS at a low-power intensity (15 mW) during a 30 s period, raster plots and PSTHs are shown in top to bottom rows in ***a*** and ***b*** at shallow lamina depths of 200–500 μm (50 μm steps). ***B***, Similar illustrations for INS-induced neural responses at a high-power intensity (60 mW). The red bar on each plot and histogram represents the irradiation time of INS. The left-hand side numbers indicate the depth from the brain surface. ***C***, Heatmap representation of INS-induced modulation effects on MUA firing rates. Horizontal and vertical axes represent stimulus intensity and depth from the brain surface, respectively. Orange–red and green–blue represent facilitative and inhibitory modulating effects of firing rates in MUAs, respectively. Data are the average from six animals (*n *= 6).

Next, we analyzed the extracellular neural activity of the mouse IC in response to INS during 30 s periods of varying stimulation intensities, ranging from 15 to 70 mW. Because the artifact of LFPs at the onset and offset of INS was relatively large, the effect of INS to LFPs was not investigated in this study (Extended Data Figs. 3-2 and 3-3). For lower stimulation intensities (≤40 mW), spontaneous firing rates were increased during stimulation (i.e., *F*_stim_/*F*_pre _> 1.0) mainly in shallow laminae (≤ 500 μm), compared with the prestimulus period (Fig. 4*A*). Typical examples of lower intensities (e.g., 15 mW), spike timings, and the corresponding spike count histograms for stimulation trials are shown in raster plots (Fig. 4*Aa*) and PSTHs (Fig. 4*Ab*), with different recording depths ranging from 200 to 500 μm in 50 μm steps; more plots of spike raster and PSTH obtained from deeper electrode sites are illustrated in Extended Data Figure 4-1. In general, firing rates increased during the stimulation period under INS: 111.2 ± 37.0 spikes/s in shallow laminae (*z *= −200 μm), 119.5 ± 23.7 spikes/s in middle laminae (*z *= −500 μm), and 176.2 ± 31.5 spikes/s in deeper laminae (*z *= −800 μm; *n *= 6)^cT^.

10.1523/ENEURO.0521-23.2024.f3-2 Figure 3-2 **INS-driven laminar responses in LFPs and MUA.** (A) In response to INS (intensity, 60  mW), local field potentials (LFPs) were recorded using a 16-channel electrode array in inferior colliculus laminae (100 to 850 μm from the brain surface in 50 μm steps). (B) Similarly, multiunit activities in inferior colliculus laminae are shown. The timings of INS onset are represented by an inverted triangle, and the duration of INS is indicated by red bars. Download Figure 3-2, TIF file.

10.1523/ENEURO.0521-23.2024.f3-3 Figure 3-3 **INS-driven laminar responses in LFPs and PSTHs (extended views of Fig. 3-2).** (A) In response to INS (intensity, 60  mW), the extended views of local field potentials (LFPs) and peri-stimulus time histogram (PSTHs) in Fig. 3-2 are shown. (B) Similarly, more extended views in Part A. The blue (a) and green (b) bars in Part B correspond to those in Part A. The depth of the recording site was 200 μm from the brain surface, which is indicated by a red arrow in Fig. 3-2A. The duration of INS is also indicated by red bars. Download Figure 3-3, TIF file.

10.1523/ENEURO.0521-23.2024.f4-1 Figure 4-1 **INS-induced modulation of neural activity in the mouse inferior colliculus.** (A) In response to infrared neural stimulation (INS) at a low power intensity (15  mW) during a 30  s period, raster plots and peristimulus time histograms (PSTHs) are shown in top to bottom rows in (a) and (b) at shallow lamina depths of 100 to 850 μm (50 μm steps). (B) Similar illustrations for INS-induced neural responses at a high power intensity (60  mW). The red bar on each plot and histogram represents the irradiation time of INS. The left-hand side numbers indicate the depth from the brain surface. Download Figure 4-1, TIF file.

In contrast, in response to near-infrared light stimulation at higher intensities (50 to 70 mW), spontaneous firing rates decreased during the stimulation period (*F*_stim_/*F*_pre _< 1.0) in all laminae (Fig. 4*B*) compared with those during the prestimulus period. In particular, inhibitory effects on firing rates were marked across laminae ranging from 100 to 700 μm: 19.6 ± 7.2 spikes/s in shallow laminae (*z *= −200 μm), 58.5 ± 28.4 spikes/s in middle laminae (*z *= −500 μm), and 78.2 ± 18.2 spikes/s in deeper laminae (*z *= −800 μm; *n *= 5)^dT^. In response to INS at intermediate intensities (30–50 mW), although the changes in the firing rate were still depth-dependent, almost no changes were observed in deeper laminae (>600 μm).

The laminar dependency of INS-induced modulation of spontaneous neural firing rate is shown in a color map representation (Fig. 4*C*). For lower stimulation intensities (15–30 mW), firing rates in surface to middle laminae (ranging from 100 to 500 μm; i.e., *z *= −500 to −100 μm) were relatively large compared with those in deep laminae (>500 μm).

**Figure 5.**
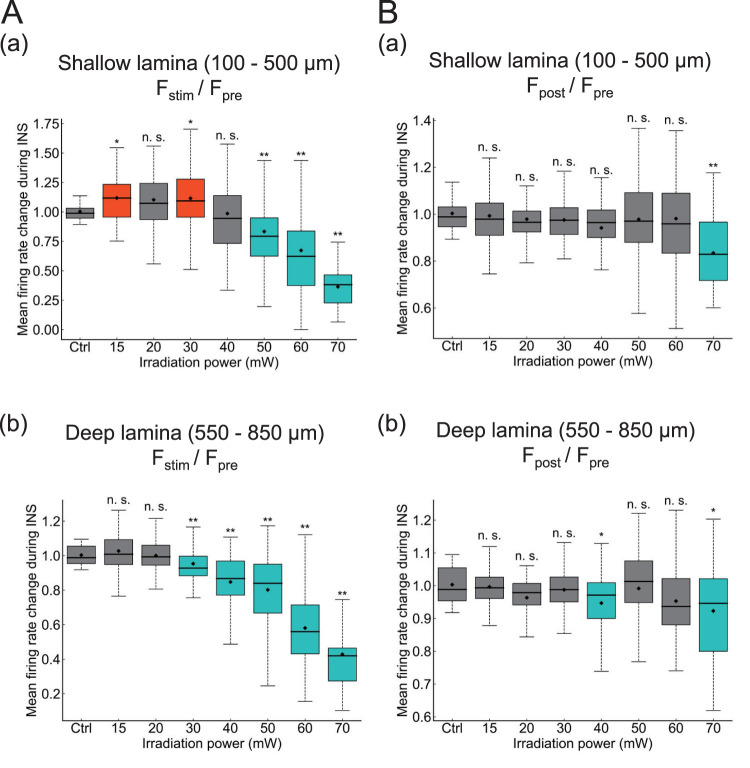
INS-induced modulation of firing rates in the mouse IC. ***A***, Ratios (*F*_stim_/*F*_pre_) of average firing rates are shown for different intensities of INS. *F*_pre_ is the average firing rate during a 20 s period before INS onset, whereas *F*_stim_ is the average firing rate during a 20 s period within a 30 s stimulation period (excluding the 5 s periods after onset and before offset). Average ratios in shallow (100–500 μm) and deep (550–850 μm) laminae are shown in ***a*** and ***b***, respectively. “Ctrl” represents the sham condition, that is, the output intensity was 0 mW. Error bars indicate standard errors of the mean. ***B***, Similarly, ratios (*F*_post_/*F*_pre_) of average firing rates are shown for different INS intensities, where *F*_post_ is the average firing rate during the 20 s period immediately after INS. Average ratios in shallow (100–500 μm) and deep (550–850 μm) laminae are shown in ***a*** and ***b***, respectively. “Ctrl” represents the sham condition. Error bars indicate standard errors of the mean. *p < 0.05, **p < 0.01, and n.s., *p *> 0.05. See Extended Data Figures 5-1 and 5-2 for more details.

Furthermore, we calculated average firing rate ratios (*F*_stim_/*F*_pre_) in shallow (100–500 μm from the surface; Fig. 5*Aa*) and deep laminae (550–850 μm; Fig. 5*Ab*) for all intensity conditions in the animals examined (*n *= 6 mice). Here, the control (sham stimulation condition) reflects the neural activity in response to INS at 0 mW intensity under the same conditions (apart from intensity). At shallow depths, average ratios were significantly larger with stimulation intensities ranging from 15 to 30 mW compared with those with the control. In contrast, average ratios with stimulation intensities ranging from 50 to 70 mW were significantly smaller compared with those with the control. Accordingly, low-power stimulation enhanced the neural activity in shallow laminae of the IC, whereas higher-power stimulations (50–70 mW) suppressed the neural activity in shallow laminae. Furthermore, in deep laminae (550–850 μm, Fig. 5*Ab*), average ratios were significantly smaller at most INS intensities (ranging from 30 to 70 mW), with no enhancement observed.

10.1523/ENEURO.0521-23.2024.f5-1 Figure 5-1 **INS-induced modulation of firing rates in the mouse inferior colliculus.** The box plots of ratios (F_post_5-20s_/F_pre_) for average firing rates are shown for different intensities of infrared neural stimulation (INS). F_pre_ is the average firing rate during a 20  s period before INS onset, whereas F_post_5-20s_ is the average firing during the 15  s, in which the immediately-after-stimulation period during 5  s is excluded to calculate the mean firing rate. Average ratios in shallow (100 to 500 μm) nd deep (550 to 850 μm) laminae in (A) and (B) are respectively shown. “Ctrl” represents the sham condition, i.e., the output intensity was 0  mW. Error bars indicate standard errors of the mean. “Ctrl” represents the sham condition. Error bars indicate standard errors of the mean. *p < 0.05, **p < 0.01, and n.s., *p *> 0.05. Download Figure 5-1, TIF file.

10.1523/ENEURO.0521-23.2024.f5-2 Figure 5-2 **Tetrodotoxin effect on spontaneous firing and INS responses in the mouse inferior colliculus.** (A) In response to infrared neural stimulation (INS), extracellular recordings of the mouse inferior colliculus (IC) (sample #1) in the absence (control, upper trace) and presence (+TTX, lower trace) of 20 μM tetrodotoxin (TTX). In (a), the INS intensity was 30  mW, and the electrode was located at a depth of 250 μm. In (b), the INS intensity was 60  mW, and the electrode was located at a 300 μm depth. A 20 μM TTX solution was directly applied to the brain surface and the neural activity evoked by INS was recorded 50  min (or more) after TTX application. No extracellular action potentials were observed in any layer in response to INS, nor spontaneous activity after TTX application. This indicates that the stimulation artifacts are small compared with INS-induced spike responses before TTX application. (B) Similar result for recordings in the IC of a different animal (sample #2). Red bars over individual waveforms indicate the duration of INS. Download Figure 5-2, TIF file.

To investigate changes in the firing rate before and after INS application, we similarly compared ratios (*F*_post_/*F*_pre_) in shallow laminae (100–500 μm from the surface) with those in the control (Fig. 5*Ba*). The ratios were significantly decreased after INS (*F*_post_/*F*_pre _< 1.0) at a stimulus power of 70 mW, yet spontaneous firing rates were unchanged at most depths, indicating that spontaneous firing rates had recovered to baseline firing after INS. Similarly, ratios (*F*_post_/*F*_pre_) in deep laminae (550–850 μm from the surface) were also compared with the control (Fig. 5*Bb*). These results showed that spontaneous firing rates were unchanged after INS at most depths with almost all INS intensities, except for 40 and 70 mW, which showed decreased firing rates.

Furthermore, because the brain surface temperature can be still raised immediately after INS, we examined ratios *F*_post_5-20s_/*F*_pre_, in which the immediately after stimulation period during 5 s is excluded to calculate the mean PSTH value of *F*_post_5-20s_. The result indicates that, for shallow IC laminae, significant differences against the sham condition (0 mW stimulation intensity) can be found under the conditions of only the two stimulation intensities: 40 and 70 mW (*p *= 0.028 and *p *= 0.0002, respectively; Extended Data Fig. 5-1). In contrast, for other stimulation intensities, there are no significant differences against the sham condition, suggesting the firing rate was recovered after the stimulation.

In addition, TTX (an antagonist of voltage-gated sodium channels) was administered to mice after an experimental series (*n *= 2; Extended Data Fig. 5-2). TTX application (20 μM) completely abolished all voltage changes in action potentials from all recording electrodes (Extended Data Fig. 5-2), confirming that extracellular voltage changes are due to the neural activity (Extended Data Fig. 5-2).

In summary, our results indicate that in response to INS, bidirectional (facilitation and suppression) modulatory effects on the neural activity were observed in the mouse IC in a manner dependent on INS intensity and recording lamina depth.

### Infrared light-driven neural modulation to sound responses in the mouse IC

**Figure 6.**
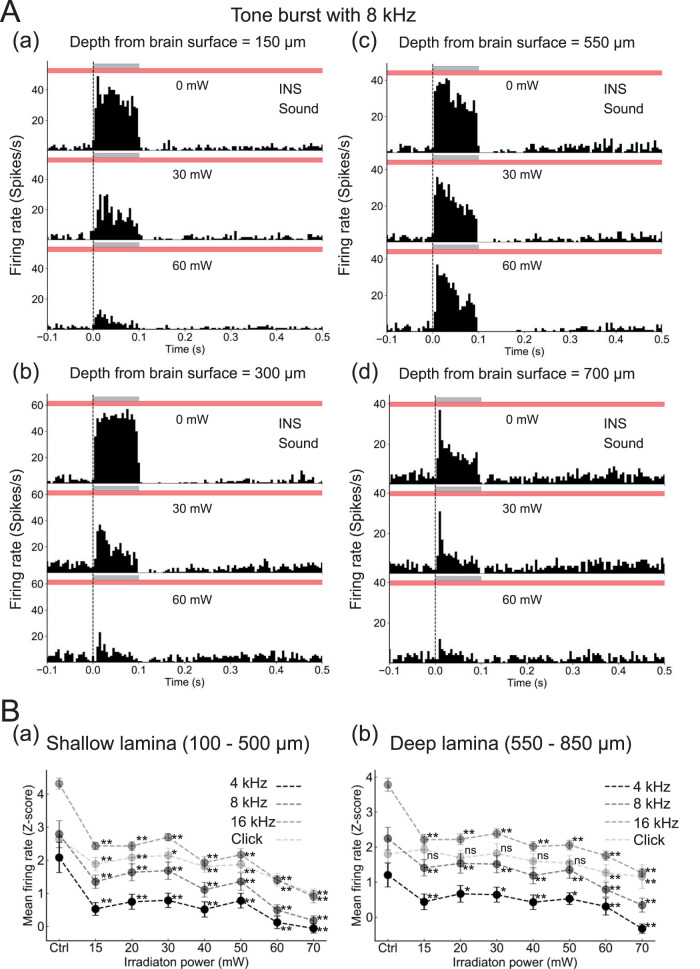
INS-induced modulation of sound-driven neural responses in the mouse IC. Sound stimuli (8 kHz tone burst with 60 dB SPL; duration, 100 ms) were presented during INS (duration, 30 s). ***A***, PSTHs at different recording laminar depths of 150, 300, 550, and 700 μm are shown in ***a–d***, respectively, for INS intensities at 0 (sham/control), 30, and 60 mW. Stimuli were repeatedly presented 20 times under the same conditions. Bars over histograms indicate the timings of sound stimulation (gray) and INS (red). ***B***, In response to INS and pure tone bursts (4, 8, and 16 kHz) or a click sound, normalized MUA firing rates (*Z* score representation) for shallow (100–500 μm) and deep (550–850 μm) laminae are shown in ***a*** and ***b***, respectively. The horizontal axis indicates the intensity of INS stimulation. “Ctrl” represents the sham condition. Error bars indicate standard errors of the mean. *p < 0.05, **p < 0.01, and n.s., *p *> 0.05. See Extended Data Figures 6-1–6-3 for more details.

To examine the modulatory effect of INS on sound-evoked responses, we presented tone bursts at a frequency of 4, 8, and 16 kHz under INS conditions and recorded sound-evoked responses (Fig. 6). These experiments were performed in response to a tone burst stimulus with a frequency of 8 kHz (60 dB SPL) and INS at different intensities (0, 30, or 60 mW). As a typical example, PSTHs obtained from firing rates at four depths (150, 300, 550, and 700 μm from the surface) are shown (Fig. 6*A*). On the basis of our previous results, we classified the IC lamina depth into two groups: (1) a shallow group at a depth ranging from 100 to 500 µm from the surface and (2) a deep group at a depth of 550–850 µm. With INS at an intensity of 60 mW, firing rates were more suppressed than at intensities of 0 and 30 mW in both shallow and deep laminae. This indicates that the modulatory effect was intensity dependent. Summary results are shown in Figure 6*B* for all stimulation intensities; normalized firing rates are represented as *Z* scores at each stimulus intensity of INS (*n *= 3 mice). Compared with the sham condition (stimulus intensity, 0 mW), sound-evoked responses during INS were significantly reduced in both shallow (Fig. 6*Ba*) and deep (Fig. 6*Bb*) lamina groups. In addition, as the stimulation intensity increased, firing rates tended to decrease in both shallow and deep lamina groups (Fig. 6*B*). Furthermore, if the sound type was a tone burst sound at a different frequency (4 and 16 kHz; Extended Data Figs. 6-1 and 6-2, respectively) or click sound (Extended Data Fig. 6-3), the overall tendency of INS-induced suppression was similar: firing rates tended to decrease in both shallow and deep lamina groups with increasing INS intensity.

10.1523/ENEURO.0521-23.2024.f6-1 Figure 6-1 **INS-induced modulation of tone burst-driven neural responses in the mouse inferior colliculus.** Spike responses to sound stimuli (4 kHz tone burst with 60  dB SPL; duration, 100  ms) presented under infrared neural stimulation (INS) (duration, 30  s). (A) Peristimulus histograms at different laminar recording depths of 150, 300, 550, and 700 μm are shown in (a), (b), (c), and (d), respectively, for INS intensities of 0 (sham), 30, and 60  mW. Stimuli were repeatedly presented 20 times under the same conditions. Bars over histograms indicate timings of sound stimulation (grey) and INS (red). (B) In response to INS and pure tone bursts (4 kHz), normalized multi-unit activity firing rates (Z-score representation) for shallow (100 to 500 μm) and deep (550 to 850 μm) laminae are shown in (a) and (b), respectively. The horizontal axis indicates the intensity of INS stimulation. *p < 0.05, **p < 0.01, and n.s., *p *> 0.05. Download Figure 6-1, TIF file.

10.1523/ENEURO.0521-23.2024.f6-2 Figure 6-2 **INS-induced modulation of tone burst-driven neural responses in the mouse inferior colliculus.** Spike responses to sound stimuli (16 kHz tone burst with 60  dB SPL; duration, 100  ms) were presented under infrared neural stimulation (INS) (duration, 30  s). (A) Peristimulus histograms at different laminar recording depths of 150, 300, 550, and 700 μm are shown in (a), (b), (c), and (d), respectively, for INS intensities at 0 (sham), 30, and 60  mW. Stimuli were repeatedly presented 20 times under the same conditions. Bars over histograms indicate timings of sound stimulation (grey) and INS (red). (B) In response to INS and pure tone bursts (16 kHz), normalized multi-unit activity firing rates (Z-score representation) for shallow (100 to 500 μm) and deep (550 to 850 μm) laminae are shown in (a) and (b), respectively. The horizontal axis indicates the intensity of INS stimulation. *p < 0.05, **p < 0.01, and n.s., *p *> 0.05. Download Figure 6-2, TIF file.

10.1523/ENEURO.0521-23.2024.f6-3 Figure 6-3 **INS-induced modulation of click-driven neural responses in the mouse inferior colliculus.** Spike responses to a click sound (60  dB SPL; duration, 0.1  ms) presented under infrared neural stimulation (INS) (duration, 30  s). (A) Peristimulus histograms at different laminar recording depths of 150, 300, 550, and 700 μm are shown in (a), (b), (c), and (d), respectively, for INS intensities of control 0 (sham), 30, and 60  mW. Stimuli were repeatedly presented 20 times under the same conditions. Bars over histograms indicate timings of sound stimulation (grey) and INS (red). (B) In response to INS and a click sound, normalized multi-unit firing rates (Z-score representation) for shallow (100 to 500 μm) and deep (550 to 850 μm) laminae are shown in (a) and (b), respectively. The horizontal axis indicates the intensity of INS stimulation. *p < 0.05, **p < 0.01, and n.s., *p *> 0.05. Download Figure 6-3, TIF file.

### Infrared light-driven modulation with an antagonist of temperature-sensitive ion channels

**Figure 7.**
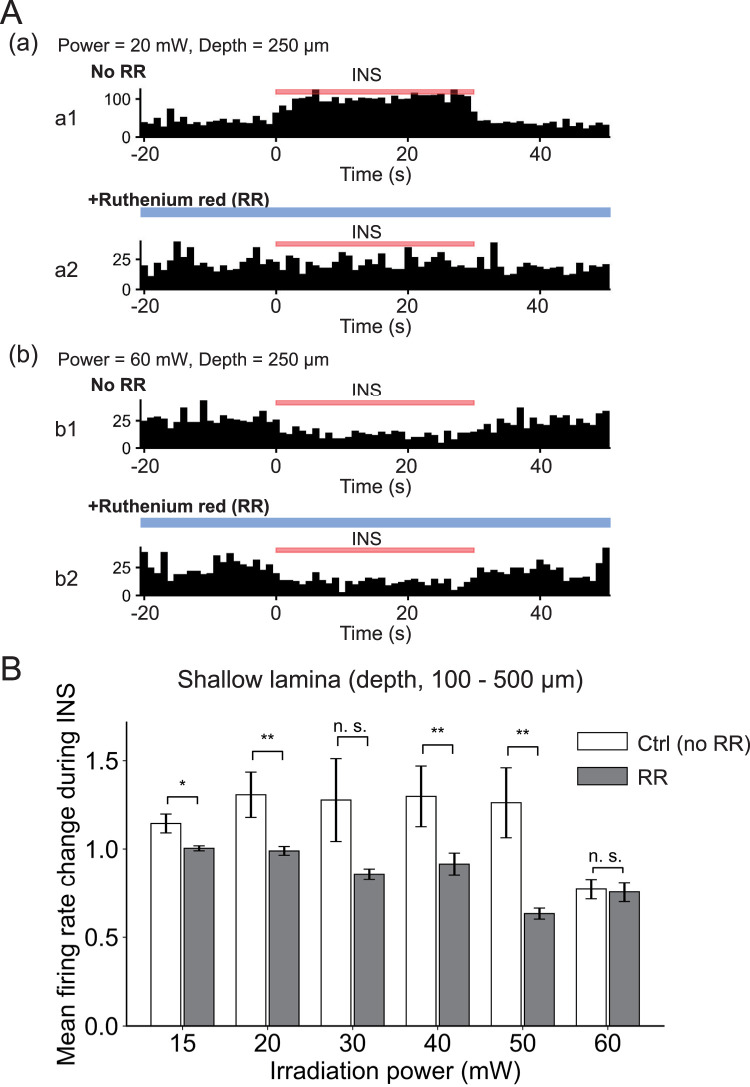
The effect of RR on INS-driven neural modulation. ***A***, Extracellular recordings of the neural activity to INS intensities of 20 mW (depth, 250 μm; ***a***) and 60 mW (depth, 250 μm; ***b***), in the absence (***a1***, ***b1***) and presence of RR (***a2***, ***b2***), an antagonist of TRPV1–4 and TRPA1 channels. Here, 20 µM RR was applied to the exposed IC surface. Bars over waveforms represent the duration (30 s) of INS (red) and continuous administration of RR (blue). ***B***, Summary of firing rations (*F*_stim_/*F*_pre_) in shallow laminae (depth, 100–500 μm; *n *= 3). *p < 0.05, **p < 0.01, and n.s., *p *> 0.05.

To examine the involvement of temperature-sensitive ion channels in the neural modulatory effect of INS, we applied RR (20 µM), as an antagonist of TRPV1–4 and TRPA1 channels, to the exposed IC surface. Before and after a sufficient infiltration period (>40 min) onto the brain surface, INS at different intensities (15–60 mW) was performed. As a typical example, Figure 7*A* shows the extracellular neural activity at stimulation intensities of 20 mW (depth, 250 μm; Fig. 7*Aa*) and 60 mW (depth, 250 μm; Fig. 7*Ab*), in the absence (top traces) and presence (bottom traces) of RR. In the presence of RR (Fig. 7*Aa2*,*b2*), the neural activity was almost suppressed during INS at lower intensities; this excludes an intensity of 30 mW (Fig. 7*B*), in which firing ratios (*F*_stim_/*F*_pre_) showed large variation between mice (*n *= 3). In summary, these results indicate that in shallow laminae (depth, 100–500 µm), average ratios are larger under non-RR conditions with stimulation intensities ranging from 15 to 50 mW (excluding 30 mW). In addition, under the RR condition, firing ratios (*F*_stim_/*F*_pre_) almost decreased (excluding 30 and 60 mW), suggesting that temperature-sensitive ion channels can influence and suppress the increase in the neural activity.

10.1523/ENEURO.0521-23.2024.f8-1 Figure 8-1 **Images of in vitro mouse brain slices after INS** Images of two mouse brain slices (thickness, 400 μm; samples #1 and #2 out of six slices) obtained in vitro following INS stimulation (power intensity, 70  mW; duration, 30  s; repetition, 5 times; inter-stimulation interval, 40  s for each preparation). The samples are mouse brain slices containing the auditory cortex in vitro. In (A) and (B), typical two preparations (#1 and #2) as representative samples are presented. In the experiments, Evans blue dye was administered from the brain surface using a thin glass pipette to indicate each site of the INS stimulation spot; the glass pipette scratch is seen in the left of Part A(b). For Parts (A) and (B), low-magnification microscopic images of brain slices in (a) and Hematoxylin & Eosin (HE) staining images in (b) are respectively shown. Download Figure 8-1, TIF file.

## Discussion

INS has the potential to serve as a valuable means of neural modulation and in the treatment of neurological disorders and brain function compensation, owing to its high spatiotemporal resolution and lower invasiveness. Here, we provide the first evidence of bidirectional (facilitatory and suppressive) modulation of the neural activity in the mouse IC induced by near-INS. We found that the modulation outcomes are dependent on stimulation intensity, laminar depth, and underlying neural activity (spontaneous activity or sound-evoked responses).

### Stimulation patterns and temperature increases

To investigate the INS-induced modulation of the neural activity, we delivered 1,940 nm CW stimulation to the IC of mice. Few studies have used CW near-INS for neural modulation (Xia and Nyberg, 2019; Horvath et al., 2020, 2022), with repetitive short and pulsed patterns applied in many studies (Wells et al., 2005; Izzo et al., 2008; Cayce et al., 2011). Pulsed wave INS lasers can repeatedly deliver heat to the brain surface over a very short period (Wells et al., 2005; Izzo et al., 2008; Schultz et al., 2012), which prevents heat from rapidly diffusing to the surrounding area. This produces a small temperature increase in only a small volume near the surface. In particular, short-pulsed INS, typically characterized by repetitive short pulses (pulse duration, <1 ms), has garnered significant attention in recent years owing to its potential neuromodulatory effects. Compared with CW INS, repetitive short-pulsed INS has two additional stimulation parameters: (1) pulse repetition frequency and (2) duty cycle associated with ratio between pulse ON and OFF times (quench period). (1) Previous studies have used various pulse repetition frequencies ranging from 2.5 to 10,000 Hz; lower frequencies (<100 Hz) have the possibility of resonance occurring between the frequency on the INS pulses and that of the brain waves (Zomorrodi et al., 2019; Shan et al., 2021). For example, Ando et al. (2011) reported that an 810 nm diode laser pulsed at 10 Hz, 100 Hz, and continuous modes, with a power density of 50 mW/cm^2^ for 12 min, was used to illuminate the head of mice with an experimental traumatic brain injury and the laser pulsed at 10 Hz was the most effective (Ando et al., 2011). This study also suggested that the 4–10 Hz rhythm occurring in the hippocampal region in the normal brain of mice could lead to positive resonance with the 10 Hz laser pulse frequency to enhance the neurorehabilitation of traumatic brain injury mice. (2) Furthermore, the major effect of quench period (pulse OFF times) in pulsed INS is known to reduce tissue heating, allowing the use of higher peak power densities than CW INS (for review, Hashmi et al., 2010). For instance, Ilic et al. (2006) compared damages in the rat brain to the laser irradiation (wavelength, 808 nm; 750 mW/cm^2^; energy density of 90 J/cm^2^) under both CW and 70 Hz pulse modes. Their result indicates that pulsed INS did not cause any damages, whereas the CW INS caused neurological deficits and histopathological damages attributed to photothermal effects.

In contrast, applying continuous (sustained) waveforms of laser irradiation at a lower-power density to the brain surface over a relatively longer period (several seconds to minutes), increases the likelihood that the heat will diffuse and penetrate deeper into the brain, resulting in relatively slower modulation of the neural activity in a wider and/or deeper brain area (Xia and Nyberg, 2019; Horvath et al., 2020, 2022). Thus, this approach is likely to be more appropriate for deeper brain stimulation, which was our expectation for this study.

To the best of our knowledge, we found only two previous reports on CW INS for neural modulation in the literature. Podgorski and Ranganathan (2016) reported that continuous illumination (250–300 mW; duration, 20 min) of laser light (*λ* = 1,040 nm) was absorbed by mouse brain tissue, with a steady-state brain temperature rise of ∼5°C (Podgorski and Ranganathan, 2016). In addition, Horvath et al. (2022) reported that a sharp-tip implantable optical microdevice used in vivo in rat somatosensory cortex, with CW infrared light (duration, 120 s; interstimulation interval, 4 min) at a laser wavelength *λ* = 1,550 nm, induced modulation of neural firing rates at 8 mW illumination. Histological experiments also demonstrated cell survival under a maximum temperature increase of 3.56°C (Horvath et al., 2022). The two studies used a longer stimulation duration than our experiments, although the wavelength and power density of INS and neural preparations were different in both studies.

### Thresholds for neural activation and tissue damage

Several researchers have numerically estimated and experimentally demonstrated a threshold for INS to the brain tissue. Using a holmium:YAG laser (wavelength *λ *= 2,120 nm; 600 mm fiber core) with a repetitive pulsed mode (350 μs pulse width; repetition rates, 2–8 Hz; duration, 10 s) and a maximum duration of 4 min, Wells et al. histologically identified a damage threshold for rat sciatic nerves in vivo. They found that radiant exposure with a lower (<1%) probability of thermal tissue damage (0.66–0.70 J/cm^2^) was significantly greater than those required for reliable stimulation to induce increased neural activity (0.34–0.48 J/cm^2^; Wells et al., 2007b).

Additionally, Goyal et al, (2012) conducted in vivo cochlear stimulation experiments using a significantly higher pulse rate of 250 Hz for a duration of 3 h (Goyal et al., 2012). They reported a functional damage threshold ranging from 25 to 40 μJ per pulse, which corresponds to an estimated average laser power of 6.25–10.0 mW. Furthermore, Chernov et al. (2014) histologically identified a damage threshold of ∼0.3–0.4 J/cm^2^ for in vivo brain tissue using a pulse rate of 200 Hz for a duration of 0.5 s (Chernov et al., 2014).

Moreover, Cayce et al. (2015) reported that pulsed infrared light (*λ* = 2,120 nm; duration, 10 s; repetition rate, 2 Hz) could be used to evoke the neural activity in human dorsal spinal roots. Here, the thermal damage was shown at a radiant exposure with a clinical holmium:YAG laser at a power density of 1.09 J/cm^2^ (Cayce et al., 2015). Ford et al. (2021) reported that infrared neural inhibition in *Aplysia* nerves was provided by irradiation (*λ* = 1,470 nm; 200 μs pulses, 200 Hz) with a laser (20 s duration; maximum pulse energy, 1.25 mJ/pulse; Ford et al., 2021).

Other previous studies have also reported a damage threshold for animals, ranging from 0.6 to 2.0 J/cm^2^: (1) 0.6 J/cm^2^ in human cortex, wavelength *λ* = 1.875 μm (Pan et al., 2023); (2) 0.6 J/cm^2^ in squirrel monkey somatosensory cortex, *λ* = 1.875 μm (Chernov et al., 2014); (3) 1.09 J/cm^2^ in human spinal cord roots, *λ* = 2.12 μm (Cayce et al., 2015); and (4) 2.0 J/cm^2^ in auditory brainstem, *λ* = 1.865 μm (Lee et al., 2009). The threshold values described above are summarized in Table 2 (Wells et al., 2007b).

**Table 2.** The threshold estimation of the brain tissue damage by INS

Wavelength in μm	Animal/human	Brain region	Damage threshold in J/cm^2^	Reference
1.875	Rat	Cortex	0.3–0.4	Chernov et al. (2014)
1.875	Squirrel monkey	Somatosensory cortex	0.6	Chernov et al. (2014)
1.875	Human	Cortex	0.6	Pan et al. (2023)
2.120	Rat	In vivo sciatic nerves	0.66–0.70	Wells et al. (2007b)
2.120	Human	Spinal cord roots	1.09	Cayce et al. (2015)
1.865	Rat	Auditory brainstem	2.0	Lee et al. (2009)

In this study, we used an INS power in the range of 15 to 70 mW. Owing to water absorption (absorption coefficient of 97.68 cm^−1^ for 1,940 nm light), the energy that reached the brain surface was reduced to ∼39% because of the saline layer (∼100 μm depth) over the brain [Eqs. (4, 6)]. If the depth of the saline layer is 100 μm and the illuminated area is assumed to cover 4,200 μm^2^, we estimated the energy density to be ∼0.870–6.12 J/cm^2^ at the brain surface. The upper limit (i.e., 6.12 J/cm^2^) of the estimated values is larger than the damage threshold (which ranges from 0.4 to 2.0 J/cm^2^; Table 2).

Therefore, our estimation suggests that lower-power densities are likely below the range of the damage threshold, whereas a higher-power density may be over the threshold. Our numerical simulation also suggests that INS-driven temperature increases in the brain tissue model are consistent with this estimation. Nevertheless, because of differences between stimulation paradigms (including wavelength, pulse width, repetitive frequency, and duration), precise quantitative comparisons with the previously described studies are difficult.

### Possible mechanisms

The brain response to INS is believed to be mainly caused by localized thermal transients generated via water and tissue absorption of near-infrared light. These near-infrared light-induced thermal transients can alter cell membrane structures, passive membrane properties, ion channel kinetics and activities, and intracellular calcium concentrations. These changes can in turn modulate cellular activities. For instance, INS studies show that brief and intense light can generate capacitive currents, owing to reversible changes in membrane dimensions and resulting in depolarization of the membrane potential (Shapiro et al., 2012; Plaksin et al., 2018). In contrast, some studies have demonstrated that potassium ion (K^+^) channels, such as voltage-dependent K^+^ channels (Ganguly et al., 2019a,b) and temperature-sensitive two–pore domain TWIK-related K^+^ channels (Zhu et al., 2022), are activated by infrared light pulses. Further, G-protein–coupled receptors are reportedly involved (Barrett et al., 2018; Moreau et al., 2018; Borrachero-Conejo et al., 2020). In addition, our result indicates that temperature-sensitive ion channels including TRPV1–4 and/or TRPA1 channels could influence and suppress the increase in the neural activity of mouse IC. However, the detailed mechanism has not yet been completely elucidated.

Our results show that the neural activity in deeper brain layers does not show a facilitatory effect at a lower-power density stimulation. A previous study reported that the distribution of gamma-aminobutyric acid (GABA) and glycine terminals is different in the IC, with the ventrolateral region of the IC core being a glycine-rich area, while the dorsomedial region is a GABA-rich area (Choy Buentello et al., 2015). These differences in laminar structure possibly dominated the modulation direction; however, specific investigation of the layer structure of the IC and modulation mechanism of INS was beyond the scope of the present work. The modulation mechanism is expected to be determined in the future by comparing the relationship between stimulation power density, laminar structure, and modulation direction of the IC and other brain regions.

### Significant/medical applications

By adjusting the intensity of the stimulation, INS has the potential to selectively induce either facilitatory or suppressive effects using a single stimulation device. In particular, the suppressive modulation effect, elicited by higher-power density stimulation across all laminae of the IC, may serve as a promising therapeutic approach for neurological disorders characterized by neural hyperactivity (such as tinnitus and hyperacusis) without concurrent brain tissue damage. Thus, our findings suggest that by adjusting the power density of the stimulation, INS has the potential to selectively induce either facilitatory or suppressive effects using a single stimulation device.

## Conclusions

Using continuous INS at a low to high-power density, we demonstrated the laminar modulation of the neural activity in the mouse IC in the presence and absence of sound. We found that the modulation effect of INS on the spontaneous neural activity was bidirectional between facilitatory and inhibitory effects in a laminar depth-dependent manner. Additionally, the modulation effect on sound-evoked responses produced only an inhibitory effect at all examined stimulus intensities over all IC laminae. Thus, this study provides important physiological evidence on the response properties of IC neurons to INS. INS can be used for the development of new therapies for neurological disorders and functional support devices for auditory central processing.

## Synthesis

Reviewing Editor: Christine Portfors, Washington State University Decisions are customarily a result of the Reviewing Editor and the peer reviewers coming together and discussing their recommendations until a consensus is reached. When revisions are invited, a fact-based synthesis statement explaining their decision and outlining what is needed to prepare a revision will be listed below. The following reviewer(s) agreed to reveal their identity: Vasiliy Tsytsarev, Hisayuki Osanai.

The reviewers agree that this study provides important considerations for future applications of infrared neural stimulation (INS). While the study was well executed, some clarifications of the methodology will improve the manuscript.

Specific comments:

The authors used tone bursts for auditory stimulation. With sound stimulation, the shape of the burst matters, since it can be made in the form of a rectangle or a more complex figure with smooth edges. This may be important because if the burst shape is not smooth, many neurons will respond to sharp corners of the burst shape. In such experimental paradigm usually the linearly increasing onset and decreasing offset of stimulus envelops set at few tens of ms. I think in this case it was not critical, but it's worth mentioning. You can cite Tsytsarev, Tanaka. Neuroreport. 2002 16;13(13):1661-6. doi: 10.1097/00001756-200209160-00019

Regarding methods: was the brain area covered with thin glass during optical imaging? If yes, this needs to be mentioned, and if not, how did the authors solve the problem of glare from uneven and wet surfaces?

The authors used TTX and RR, in a certain concentration and in a certain way, both methods require reference ##

Minor criticism:

The authors write "near-infrared" using wavelength is 1940 nm. Possible, it would be more correct to call it infrared, please check the terminology.

The presentation of a subject is systematic and comprehensive and analysis is proper. I am happy to recommend the manuscript for the publication after minor corrections mentioned above.

Continuous stim vs. Pulsed stim:

- The authors argue that continuous light stimulation has an advantage than pulsed stimulation in deep stimulation with less neuronal damage because the heat is expected to penetrate deeper easily. Are there any reports supporting this expectation? If not, could the authors also simulate effect of repetitive INS on brain temperature? Although I basically agree with the authors' expectation, the readers may think another possibility that short-pulse stimulation may less damage to neurons since its effect is instant and neurons can recover during intervals between stimulation pulses. The authors' argument would be more convincing if it is demonstrated that repetitive short pulses need to generate more heat to provide heat at deeper layer. Discussing the above possibility would be also helpful for reader's understanding.

Neuronal damage:

- Although the neuronal damage of continuous stimulation used in this study is suggested to be non-severe or ignorable because post-stim spontaneous firing rates are mostly recovered to the pre-stim level, still there is a possibility to cause potential tissue damage as discussed. If possible, can the authors investigate cell-damage with histology? Or at least, please discuss which intensity was expected to be safe.

- Fig 5B shows firing rate does not fully recover after 70mW stim. This is reasonable considering the surface brain temperature is still raised immediately after stimulation end as shown in Fig 2Ac, because Fpost is average firing rate including immediately after stimulation. Does the firing rate recovered to the baseline level if the immediately-after-stimulation epoch is excluded?

- While the tissue damage is discussed with the unit J/cm2, Fig. 2 demonstrated only with temperature. Could the authors also demonstrate light energy distribution in a brain with the same unit?

Other major comments:

- In p15, "Further, an irradiation power of 50 mW caused a temperature rise of 2℃ at a depth of 0.4 mm (z = −0.4 mm) from the surface, resulting in the possibility of modulating neural firing in layers 3/4 of mouse brain tissue (Figure 2Ba, b).": Can the authors explain or add supportive references why a temperature rise of 2℃ is expected to modulate neural firing?

- Although the authors focused on continuous light stimulation in this manuscript, could the authors briefly summarize what is known about effects of pulsed INS to clarify the impact of this study for readers who are unfamiliar with INS?

- Please add references how the authors determined each parameter of computational model described in Table 1.

Minor comments:

- In Fig 2Ac, x label "Time (ms)" is typo of "Time (s)".

- Image resolutions of the raster plots are low, please use higher dpi.

- While the authors showed LFP response to sound in Fig. 3, the effect of INS to LFP is not investigated in the manuscript. Were there any reasons, e.g. photovoltaic artifact hampered this investigation?

- Fig. 4AB show the effect of INS only until 500um depth. Can the authors show spike raster and PSTH of deeper electrode sites too?

- In p6 "Near-infrared light stimulation setup" section, "The output of the laser module was controlled by a function generator (WF1947; NF Corp., Yokohama, Japan), which fed a trigger signal for a pulse train to the module": A word "a pulse train" would be confusing because the authors used continuous stimulation but not pulsed wave. Please use other words, e.g. fed a trigger signal for generating laser output, etc.

- It is helpful if the authors can align x-axis of LFPs and MUAs in Fig.3 for readers to easily compare temporal dynamics of LFPs and spike activities.
