# Supplementary figures and images for: Modulatory Effects on Laminar Neural Activity Induced by Near-Infrared Light Stimulation with a Continuous Waveform to the Mouse Inferior Colliculus In Vivo
Source: eNeuro. 2024 May 7;11(5):ENEURO.0521-23.2024. doi: 10.1523/ENEURO.0521-23.2024 (PMC11091952; doi:10.1523/ENEURO.0521-23.2024)

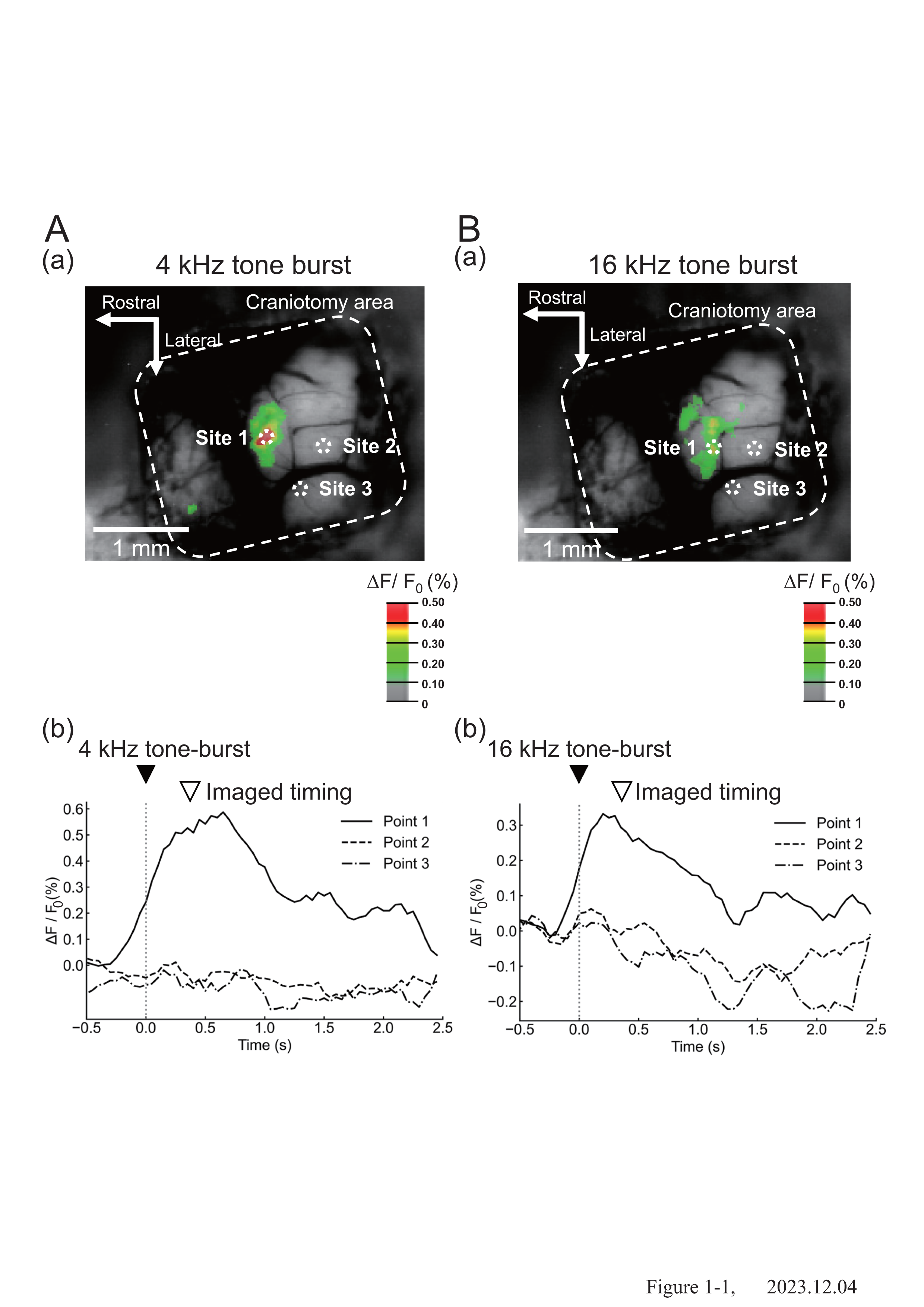

Supplement: Figure 1-1 — Flavoprotein autofluorescence imaging. (A) Flavoprotein autofluorescence imaging to locate sound-driven responses in the mouse inferior colliculus in (a). In response to a 4 kHz tone burst stimulus, time courses of flavoprotein imaging in several positions (sites 1 to 3) of activated areas in (a) are illustrated in (b). (B) Similarly, in response to a 16 kHz tone burst stimulus. Timings of sound onset and imaged time points are represented by black (▾) and white (▽) inverted triangles, respectively. Download Figure 1-1, TIF file. [file eneuro-11-ENEURO.0521-23.2024-s006.tif]

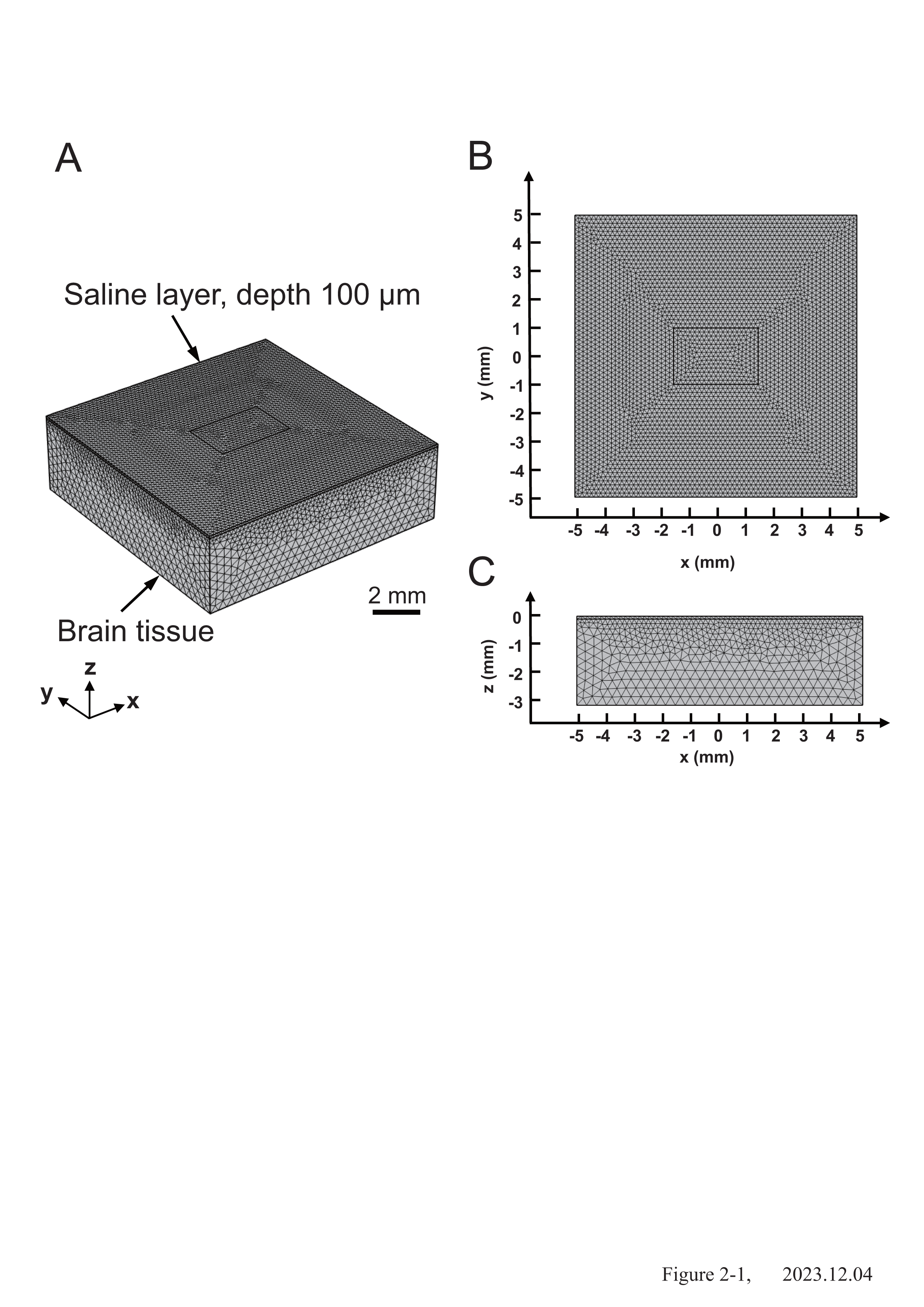

Supplement: Figure 2-1 — Mesh structure of a three-dimensional brain tissue model Three-dimensional (3D) structure for finite element method (FEM) modeling to numerically obtain temperature changes in the brain tissue model. For simplicity, an optical fiber is not drawn, but has a tilt angle of 40° to the brain surface. The 3D structure of the brain tissue model in (A) and projection to two-dimensional planes; x-y plane projection in (B) and x-z projection in (C). There is a saline layer on top, with a depth of 100 μm. Download Figure 2-1, TIF file. [file eneuro-11-ENEURO.0521-23.2024-s007.tif]

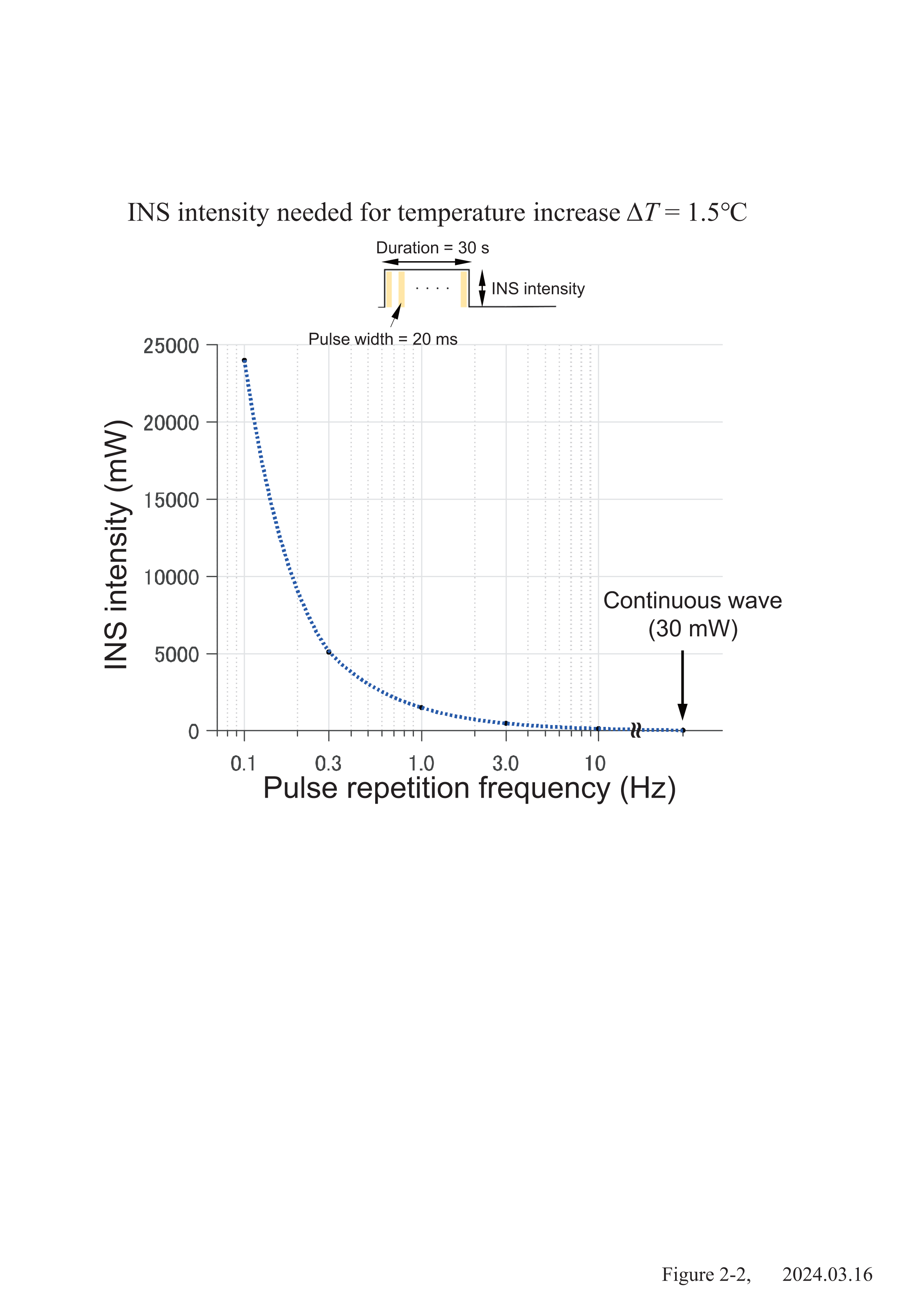

Supplement: Figure 2-2 — INS intensities of repetitive pulsed stimulation and continuous wave stimulation. For repetitive pulsed stimulation, intensities need to provide the same heat (temperature increase ΔT = 1.5℃) at deeper layer (800 μm from the brain surface) were numerically calculated when the repetition rate was one of 0.1, 0.3, 1.0, 3.0, and 10 Hz. In the repetitive pulsed stimulation, the pulse width was 20 ms, and the stimulation duration was 30 s. In the continuous wave light stimulation (the rightmost point indicated by an arrow), similarly, the stimulation duration was 30 s and the layer was 800 μm from the brain surface. In the case, INS intensity was 30 mW. Download Figure 2-2, TIF file. [file eneuro-11-ENEURO.0521-23.2024-s008.tif]

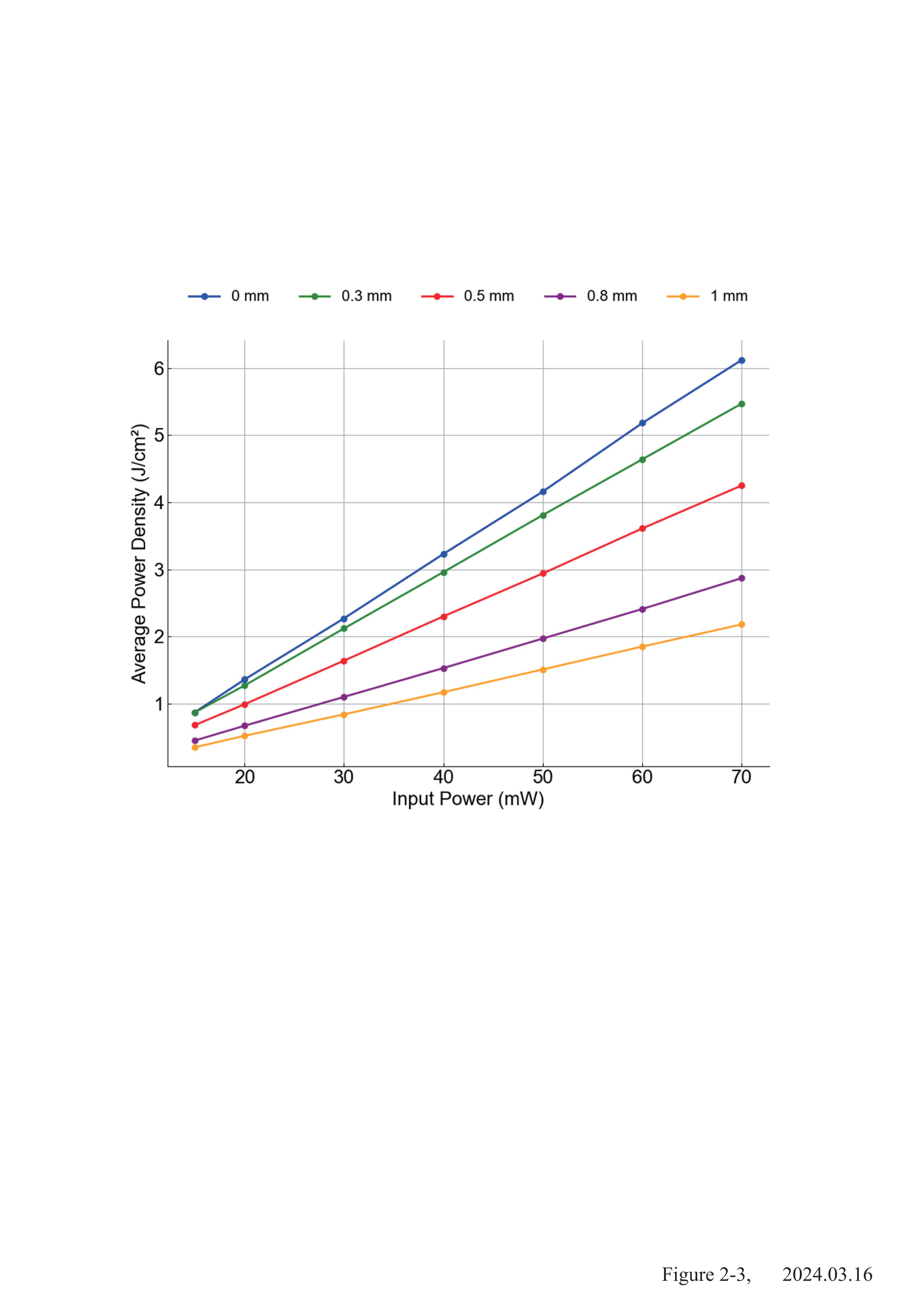

Supplement: Figure 2-3 — Light energy distribution in a brain with the unit of J/cm2. Numerical simulation result of energy density (unit, J/cm2) as the function of the input powers (15, 20, 30, 40, 50, 60, and 70 mW) 30 s after INS onset, whose changes depended on the distance from the brain surface: 0, 0.3, 0.5, 0.8, and 1.0 mm. The INS stimulation duration was 30 s. Download Figure 2-3, TIF file. [file eneuro-11-ENEURO.0521-23.2024-s009.tif]

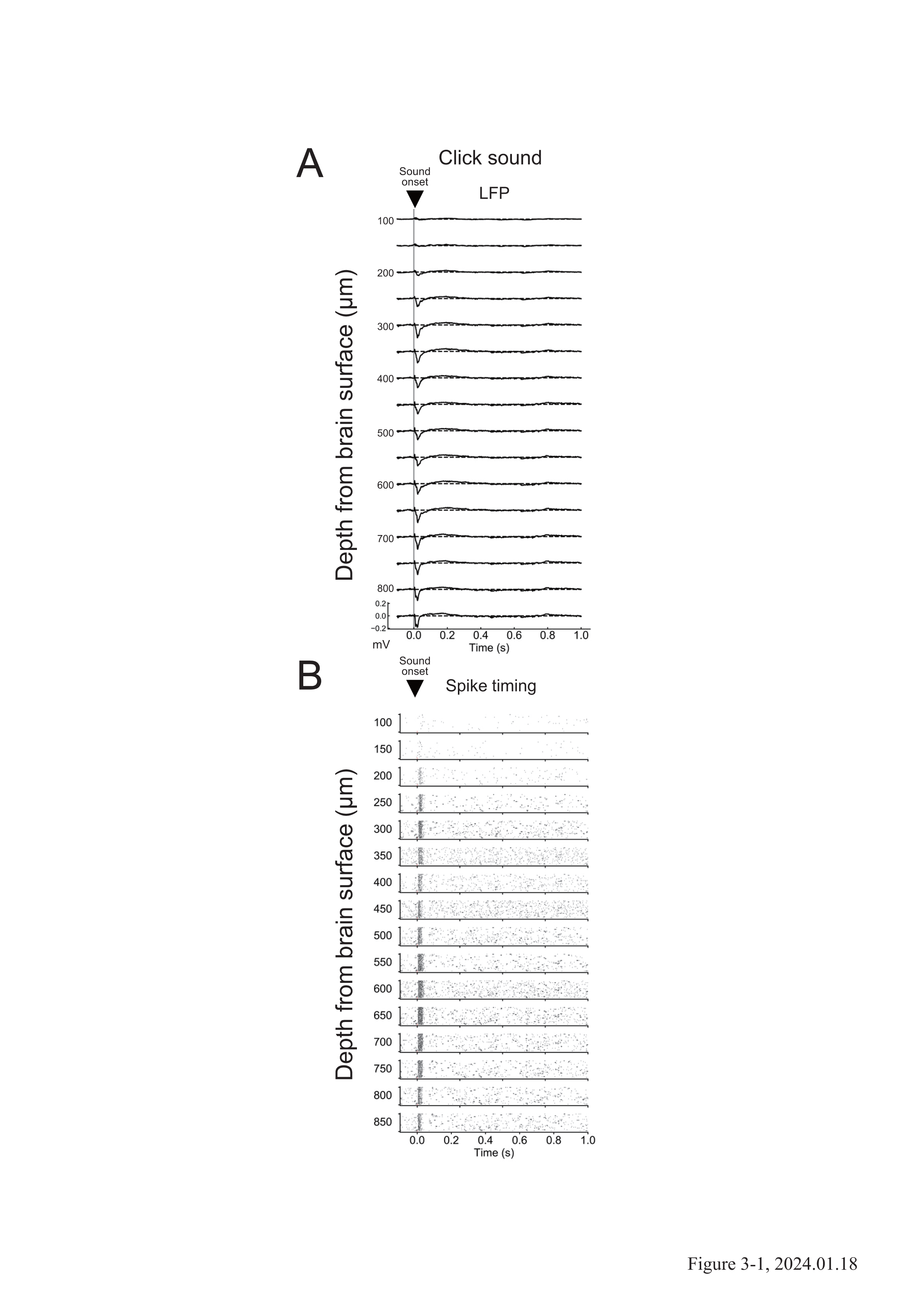

Supplement: Figure 3-1 — Sound-driven laminar responses to a click sound in the mouse inferior colliculus. (A) In response to click sound stimulation (60 dB SPL; and duration, 0.1 ms), local field potentials (LFPs) were recorded using a 16-channel electrode array in inferior colliculus laminae (100 to 850 μm from the brain surface in 50 μm steps). The timing of sound onset is represented by an inverted triangle. (B) Raster plots of multi-unit activity in response to click sound stimulation are shown, and correspond to the same experimental trials in (A). In each raster plot, 20 representations of responses to a click sound are shown; the depth from the surface is indicated by numbers (100 to 850 in μm) on the left-hand side. The timing of sound onset is represented by an inverted triangle. Download Figure 3-1, TIF file. [file eneuro-11-ENEURO.0521-23.2024-s010.tif]

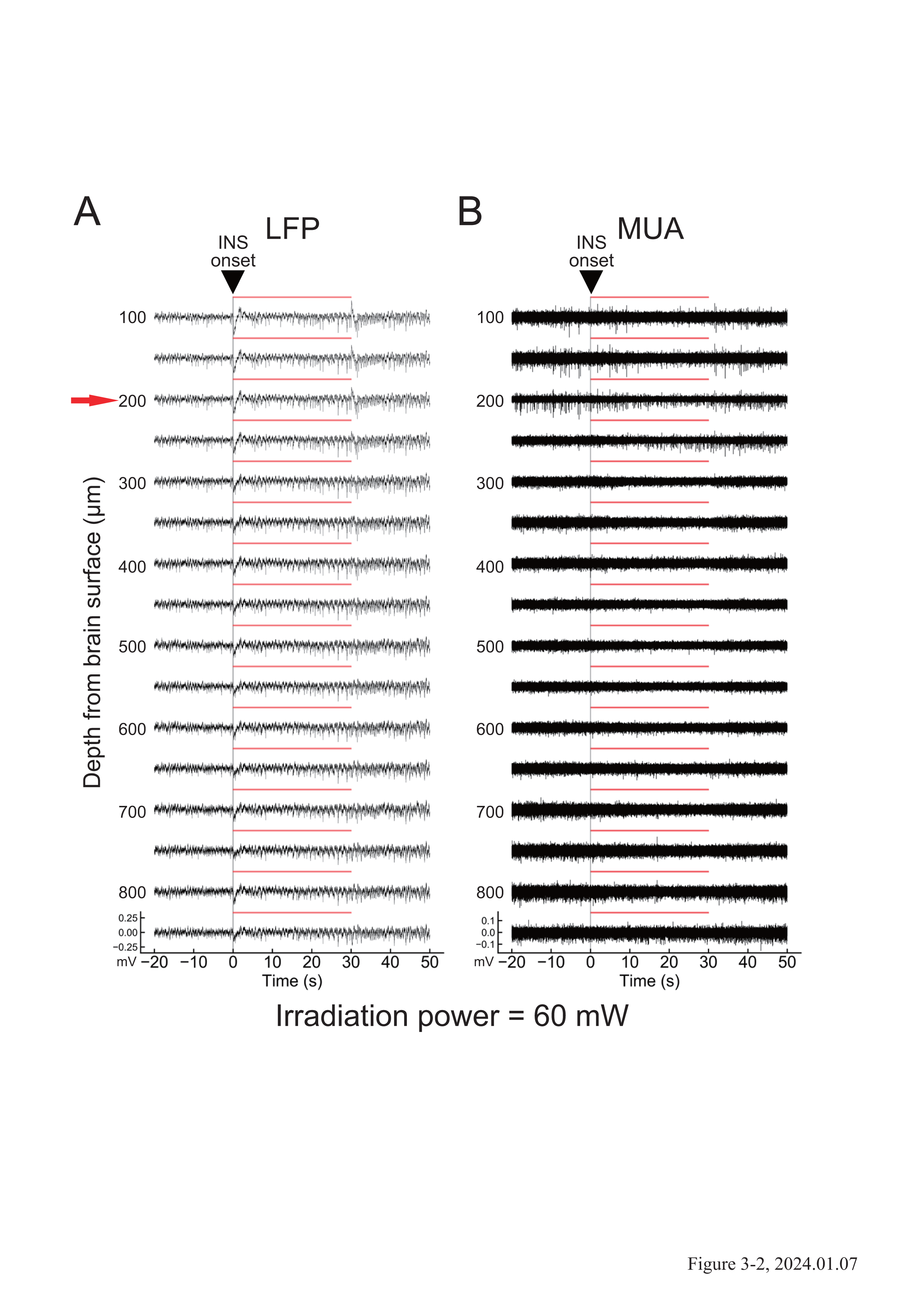

Supplement: Figure 3-2 — INS-driven laminar responses in LFPs and MUA. (A) In response to INS (intensity, 60 mW), local field potentials (LFPs) were recorded using a 16-channel electrode array in inferior colliculus laminae (100 to 850 μm from the brain surface in 50 μm steps). (B) Similarly, multiunit activities in inferior colliculus laminae are shown. The timings of INS onset are represented by an inverted triangle, and the duration of INS is indicated by red bars. Download Figure 3-2, TIF file. [file eneuro-11-ENEURO.0521-23.2024-s011.tif]

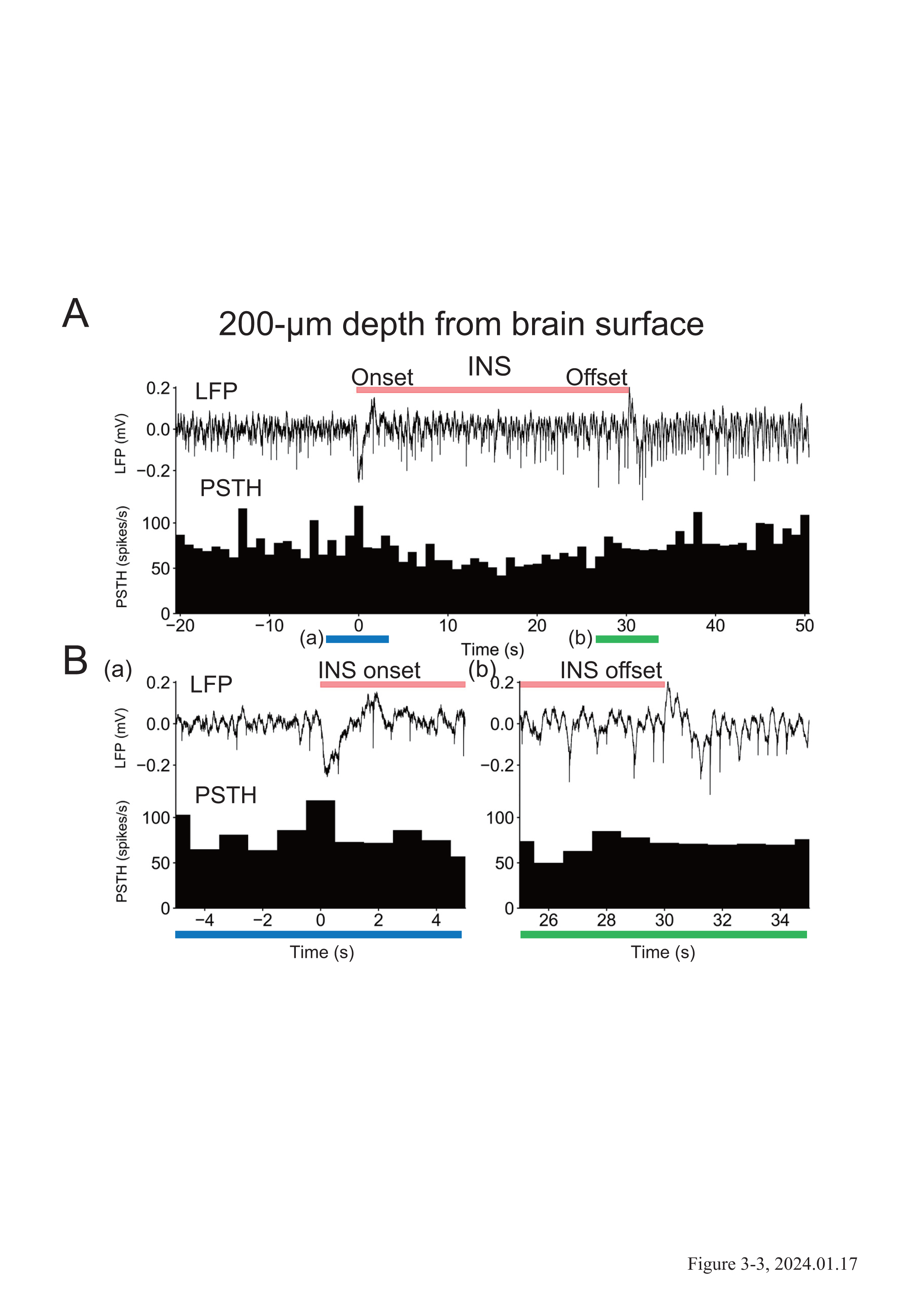

Supplement: Figure 3-3 — INS-driven laminar responses in LFPs and PSTHs (extended views of Fig. 3-2). (A) In response to INS (intensity, 60 mW), the extended views of local field potentials (LFPs) and peri-stimulus time histogram (PSTHs) in Fig. 3-2 are shown. (B) Similarly, more extended views in Part A. The blue (a) and green (b) bars in Part B correspond to those in Part A. The depth of the recording site was 200 μm from the brain surface, which is indicated by a red arrow in Fig. 3-2A. The duration of INS is also indicated by red bars. Download Figure 3-3, TIF file. [file eneuro-11-ENEURO.0521-23.2024-s012.tif]

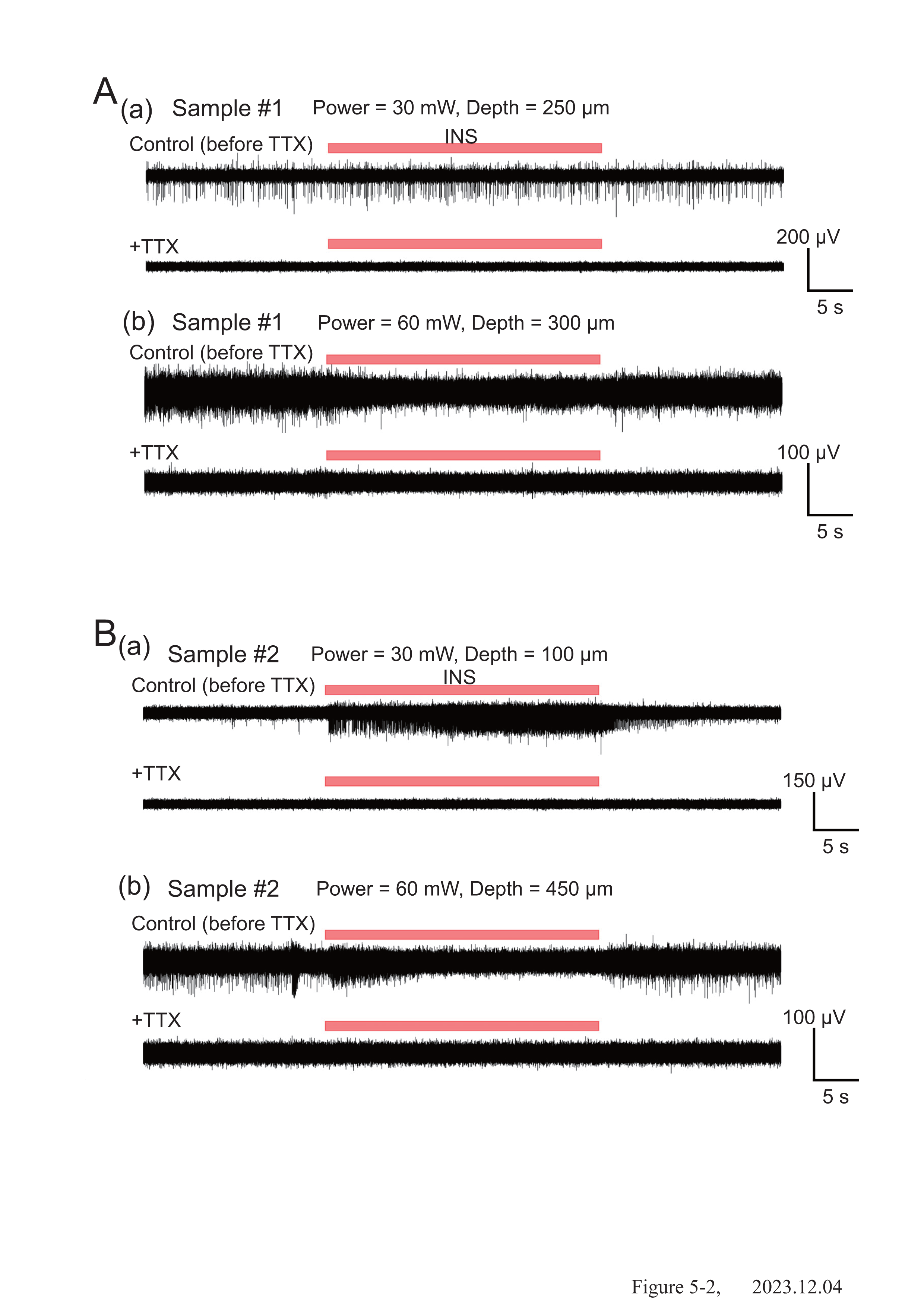

Supplement: Figure 4-1 — INS-induced modulation of neural activity in the mouse inferior colliculus. (A) In response to infrared neural stimulation (INS) at a low power intensity (15 mW) during a 30 s period, raster plots and peristimulus time histograms (PSTHs) are shown in top to bottom rows in (a) and (b) at shallow lamina depths of 100 to 850 μm (50 μm steps). (B) Similar illustrations for INS-induced neural responses at a high power intensity (60 mW). The red bar on each plot and histogram represents the irradiation time of INS. The left-hand side numbers indicate the depth from the brain surface. Download Figure 4-1, TIF file. [file eneuro-11-ENEURO.0521-23.2024-s013.tif]

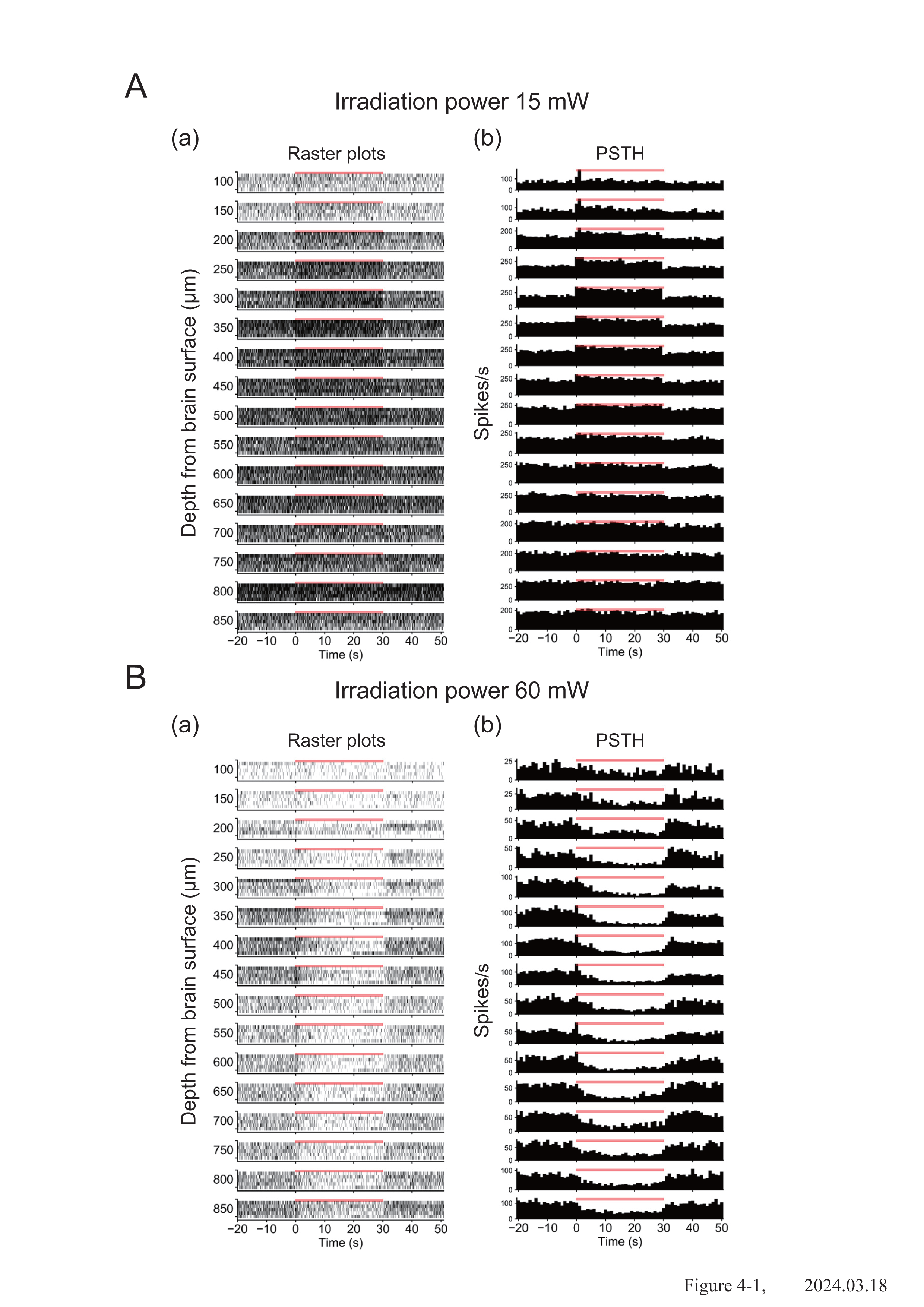

Supplement: Figure 5-1 — INS-induced modulation of firing rates in the mouse inferior colliculus. The box plots of ratios (Fpost_5-20s/Fpre) for average firing rates are shown for different intensities of infrared neural stimulation (INS). Fpre is the average firing rate during a 20 s period before INS onset, whereas Fpost_5-20s is the average firing during the 15 s, in which the immediately-after-stimulation period during 5 s is excluded to calculate the mean firing rate. Average ratios in shallow (100 to 500 μm) nd deep (550 to 850 μm) laminae in (A) and (B) are respectively shown. “Ctrl” represents the sham condition, i.e., the output intensity was 0 mW. Error bars indicate standard errors of the mean. “Ctrl” represents the sham condition. Error bars indicate standard errors of the mean. *p < 0.05, **p < 0.01, and n.s., p > 0.05. Download Figure 5-1, TIF file. [file eneuro-11-ENEURO.0521-23.2024-s014.tif]

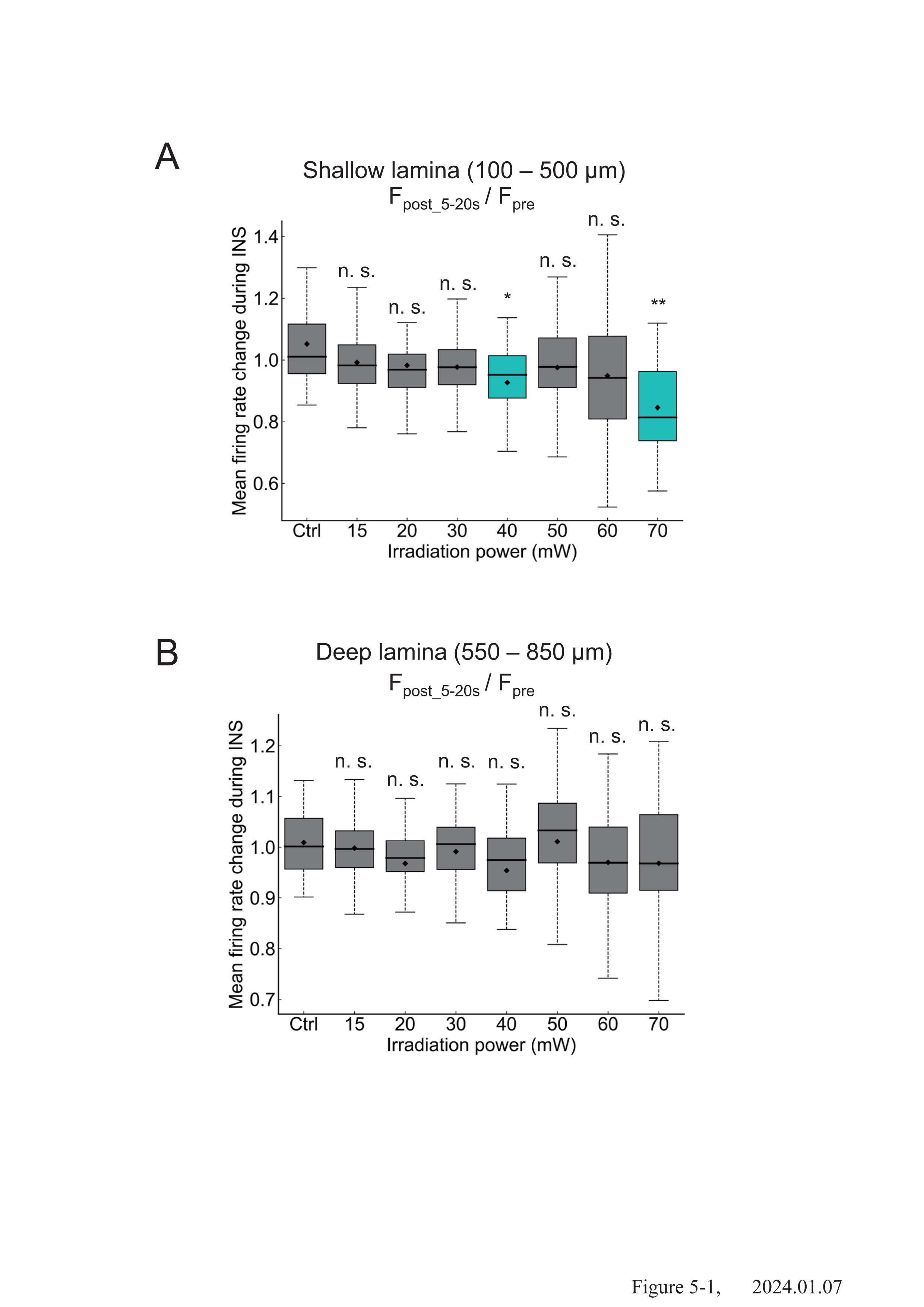

Supplement: Figure 5-2 — Tetrodotoxin effect on spontaneous firing and INS responses in the mouse inferior colliculus. (A) In response to infrared neural stimulation (INS), extracellular recordings of the mouse inferior colliculus (IC) (sample #1) in the absence (control, upper trace) and presence (+TTX, lower trace) of 20 μM tetrodotoxin (TTX). In (a), the INS intensity was 30 mW, and the electrode was located at a depth of 250 μm. In (b), the INS intensity was 60 mW, and the electrode was located at a 300 μm depth. A 20 μM TTX solution was directly applied to the brain surface and the neural activity evoked by INS was recorded 50 min (or more) after TTX application. No extracellular action potentials were observed in any layer in response to INS, nor spontaneous activity after TTX application. This indicates that the stimulation artifacts are small compared with INS-induced spike responses before TTX application. (B) Similar result for recordings in the IC of a different animal (sample #2). Red bars over individual waveforms indicate the duration of INS. Download Figure 5-2, TIF file. [file eneuro-11-ENEURO.0521-23.2024-s015.tif]

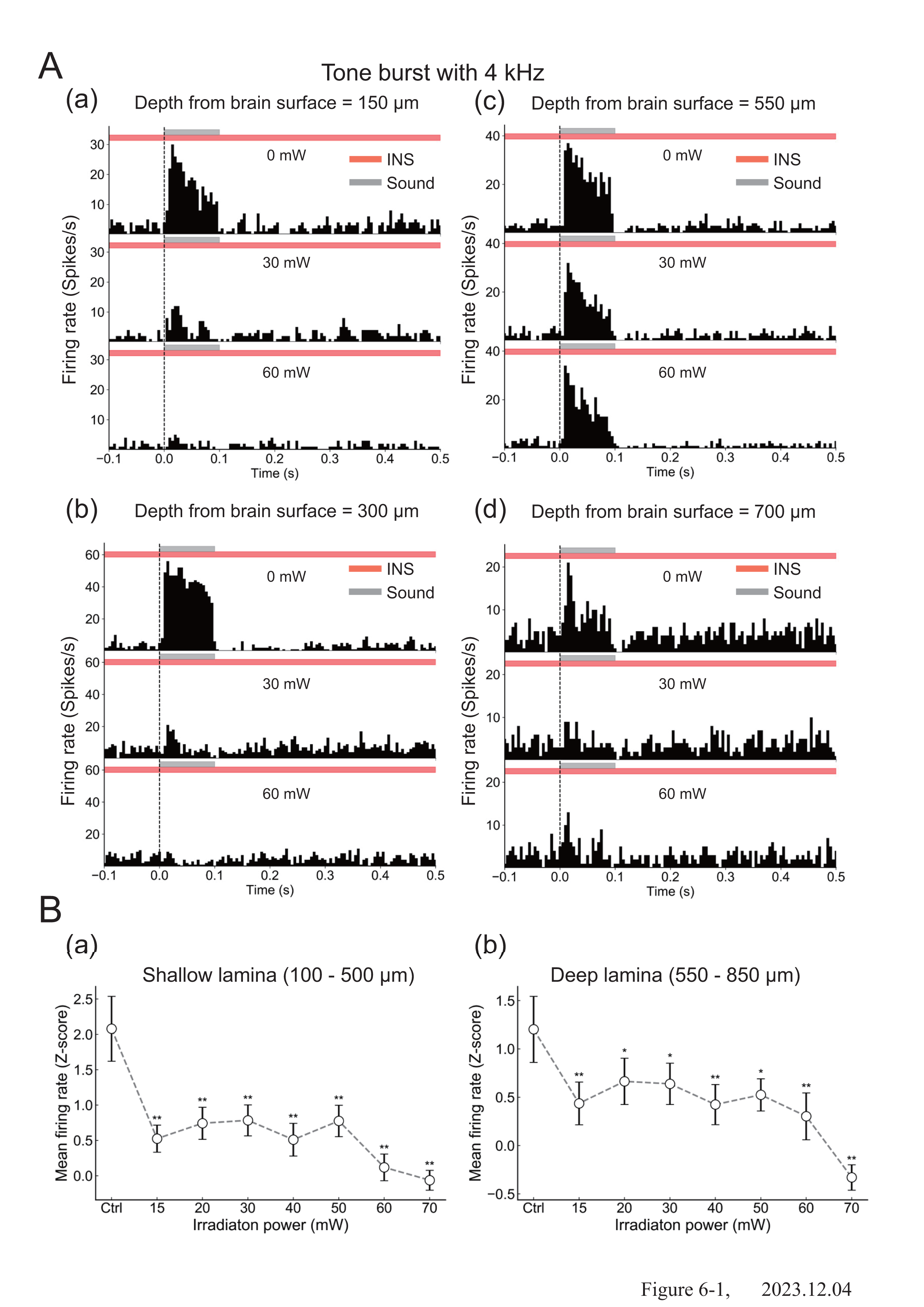

Supplement: Figure 6-1 — INS-induced modulation of tone burst-driven neural responses in the mouse inferior colliculus. Spike responses to sound stimuli (4 kHz tone burst with 60 dB SPL; duration, 100 ms) presented under infrared neural stimulation (INS) (duration, 30 s). (A) Peristimulus histograms at different laminar recording depths of 150, 300, 550, and 700 μm are shown in (a), (b), (c), and (d), respectively, for INS intensities of 0 (sham), 30, and 60 mW. Stimuli were repeatedly presented 20 times under the same conditions. Bars over histograms indicate timings of sound stimulation (grey) and INS (red). (B) In response to INS and pure tone bursts (4 kHz), normalized multi-unit activity firing rates (Z-score representation) for shallow (100 to 500 μm) and deep (550 to 850 μm) laminae are shown in (a) and (b), respectively. The horizontal axis indicates the intensity of INS stimulation. *p < 0.05, **p < 0.01, and n.s., p > 0.05. Download Figure 6-1, TIF file. [file eneuro-11-ENEURO.0521-23.2024-s016.tif]

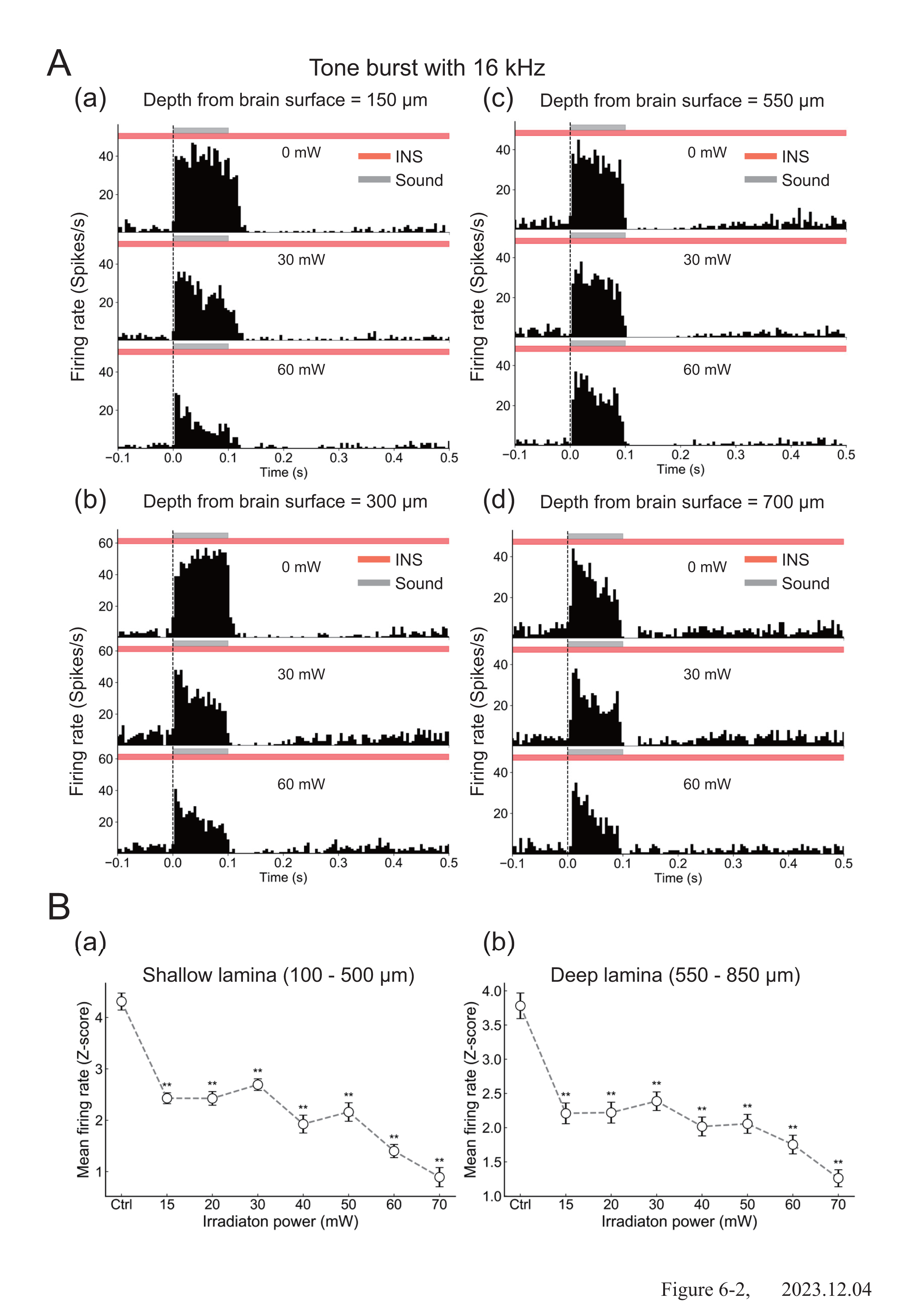

Supplement: Figure 6-2 — INS-induced modulation of tone burst-driven neural responses in the mouse inferior colliculus. Spike responses to sound stimuli (16 kHz tone burst with 60 dB SPL; duration, 100 ms) were presented under infrared neural stimulation (INS) (duration, 30 s). (A) Peristimulus histograms at different laminar recording depths of 150, 300, 550, and 700 μm are shown in (a), (b), (c), and (d), respectively, for INS intensities at 0 (sham), 30, and 60 mW. Stimuli were repeatedly presented 20 times under the same conditions. Bars over histograms indicate timings of sound stimulation (grey) and INS (red). (B) In response to INS and pure tone bursts (16 kHz), normalized multi-unit activity firing rates (Z-score representation) for shallow (100 to 500 μm) and deep (550 to 850 μm) laminae are shown in (a) and (b), respectively. The horizontal axis indicates the intensity of INS stimulation. *p < 0.05, **p < 0.01, and n.s., p > 0.05. Download Figure 6-2, TIF file. [file eneuro-11-ENEURO.0521-23.2024-s017.tif]

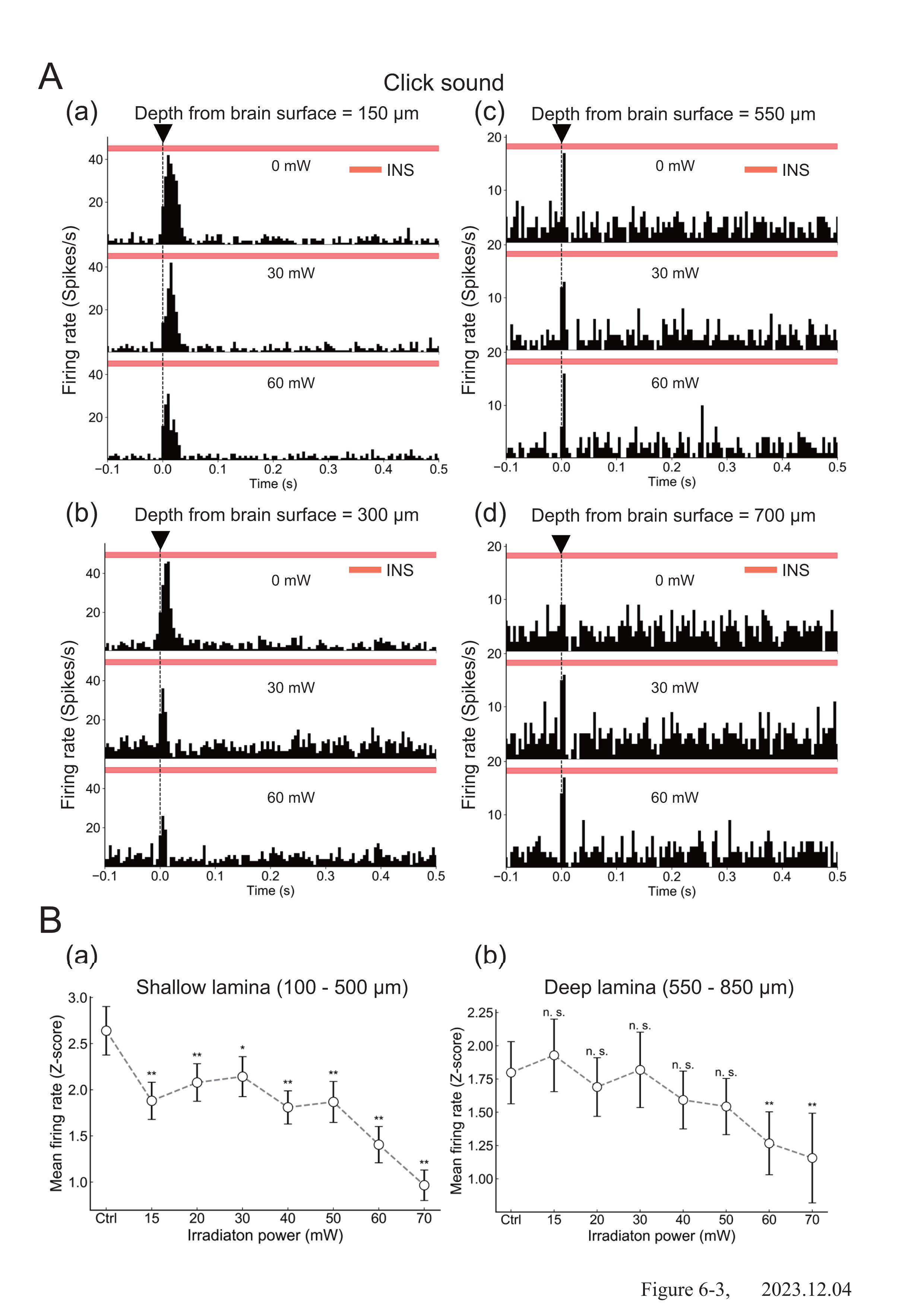

Supplement: Figure 6-3 — INS-induced modulation of click-driven neural responses in the mouse inferior colliculus. Spike responses to a click sound (60 dB SPL; duration, 0.1 ms) presented under infrared neural stimulation (INS) (duration, 30 s). (A) Peristimulus histograms at different laminar recording depths of 150, 300, 550, and 700 μm are shown in (a), (b), (c), and (d), respectively, for INS intensities of control 0 (sham), 30, and 60 mW. Stimuli were repeatedly presented 20 times under the same conditions. Bars over histograms indicate timings of sound stimulation (grey) and INS (red). (B) In response to INS and a click sound, normalized multi-unit firing rates (Z-score representation) for shallow (100 to 500 μm) and deep (550 to 850 μm) laminae are shown in (a) and (b), respectively. The horizontal axis indicates the intensity of INS stimulation. *p < 0.05, **p < 0.01, and n.s., p > 0.05. Download Figure 6-3, TIF file. [file eneuro-11-ENEURO.0521-23.2024-s018.tif]

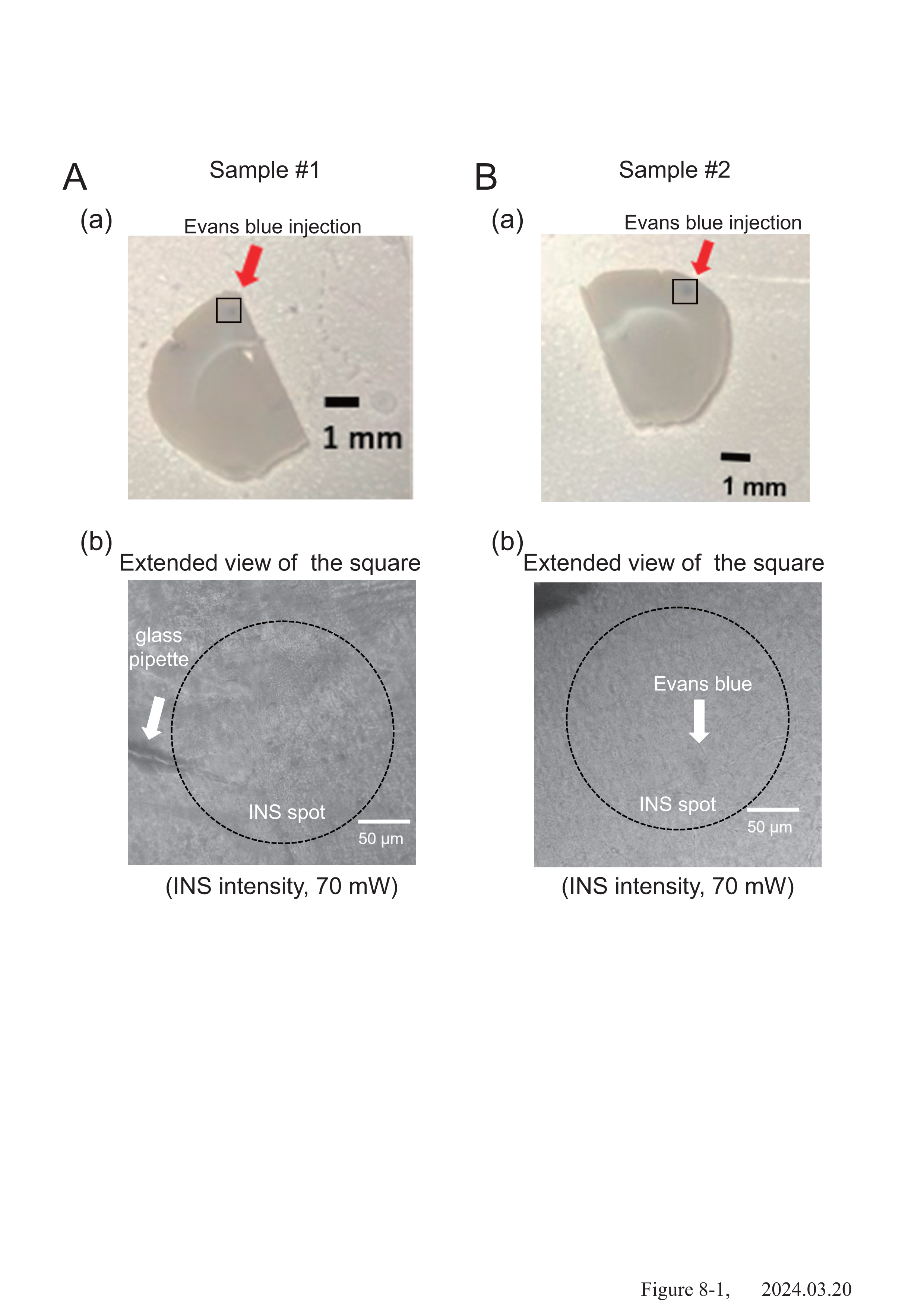

Supplement: Figure 8-1 — Images of in vitro mouse brain slices after INS Images of two mouse brain slices (thickness, 400 μm; samples #1 and #2 out of six slices) obtained in vitro following INS stimulation (power intensity, 70 mW; duration, 30 s; repetition, 5 times; inter-stimulation interval, 40 s for each preparation). The samples are mouse brain slices containing the auditory cortex in vitro. In (A) and (B), typical two preparations (#1 and #2) as representative samples are presented. In the experiments, Evans blue dye was administered from the brain surface using a thin glass pipette to indicate each site of the INS stimulation spot; the glass pipette scratch is seen in the left of Part A(b). For Parts (A) and (B), low-magnification microscopic images of brain slices in (a) and Hematoxylin & Eosin (HE) staining images in (b) are respectively shown. Download Figure 8-1, TIF file. [file eneuro-11-ENEURO.0521-23.2024-s019.tif]
