# Supplementary material for: Modulatory Effects on Laminar Neural Activity Induced by Near-Infrared Light Stimulation with a Continuous Waveform to the Mouse Inferior Colliculus In Vivo
Source: eNeuro. 2024 May 7;11(5):ENEURO.0521-23.2024. doi: 10.1523/ENEURO.0521-23.2024 (PMC11091952; doi:10.1523/ENEURO.0521-23.2024)
Supplement: Extended Data — Download Extended Data, ZIP file. [file eneuro-11-ENEURO.0521-23.2024-s005.zip › Brief_report_eNeuro_INS_CW_No_results.docx]

[
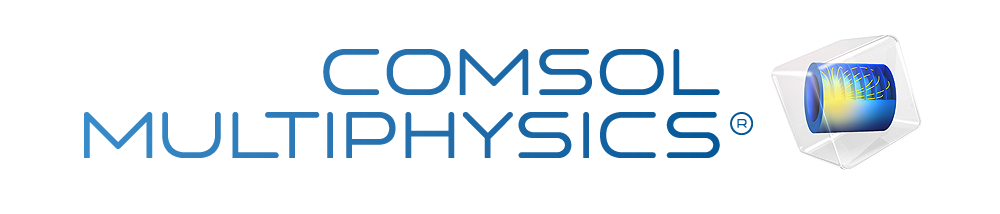
](https://www.comsol.com/)

eNeuro INS CW

Report date

Dec 8, 2023, 1:48:09 PM

Contents

[1. Global Definitions](#cs8613314)

[1.1. Parameters](#cs6380649)

[1.2. Variables](#cs3834207)

[1.3. Functions](#cs2405768)

[2. Component 1](#cs7034117)

[2.1. Definitions](#cs2127133)

[2.2. Geometry 1](#cs4908884)

[2.3. Materials](#cs4411987)

[2.4. Bioheat Transfer](#cs5321369)

[2.5. Events](#cs6923918)

[2.6. Mesh 2](#cs3416859)

[3. Study 1](#cs2543496)

[3.1. Parametric Sweep](#cs2818256)

[3.2. Time Dependent](#cs6207850)

No information about results

1. Global Definitions

| Date | Dec 2, 2023, 2:28:24 PM |
| --- | --- |

Global settings

| Name | ENeuro INS CW.mph |
| --- | --- |
| Path | /lustre0/home/m22136/Students/Author_tmp/eNeuro_INS_CW.mph |
| Version | COMSOL Multiphysics 6.1 (Build: 346) |

Used products

| COMSOL Multiphysics |
| --- |
| Heat Transfer Module |

Computer information

| CPU | Intel(R) Xeon(R) Gold 6130 CPU @ 2.10GHz, 12 sockets, 50 cores, 754.83 GB RAM |
| --- | --- |
| Operating system | Linux |

- 1. Parameters

Parameters of bioheat transfer by laser

| **Name** | **Expression** | **Value** | **Description** |
| --- | --- | --- | --- |
| Qs | 70[mW] | 0.07 W |  |
| mc | 0.8 | 0.8 |  |
| mu_a | 119.83[1/cm] | 11983 1/m | absorption coefficient of water in 1980nm |
| rho_b | 1057[kg/m^3] | 1057 kg/m³ | blood density |
| Cp_b | 3600[J/(kg*K)] | 3600 J/(kg·K) | blood heat capacity |
| omega_b | 0.012[1/s] | 0.012 1/s | blood perfusion |
| Tb | 36.7[degC] | 309.85 K | blood temperature |
| Q0 | 10[mW] | 0.01 W | laser power |
| Qmet | 13698[W/m^3] | 13698 W/m³ | Metabolic heat |
| NA | 0.22 | 0.22 | number of NA |
| nb | 1.36 | 1.36 | refractive index of gray matter |
| nw | 1.33 | 1.33 | refractive index of water |
| Rc | 0 | 0 | refrective index of brain |
| ts | 70[s] | 70 s | simulation time |
| td_h | 50[s] | 50 s | time to heat damage |
| rho | 1040[kg/m^3] | 1040 kg/m³ | tissue density |
| Cp | 3650[J/(kg*K)] | 3650 J/(kg·K) | tissue heat capacity |
| Tinit | 37[degC] | 310.15 K | tissue temperature |
| k | 0.527[W/(m*K)] | 0.527 W/(m·K) | tissue thermal conductivity |

- 1. Variables
     1. variable of bioheat transfer by laser

Selection

| Geometric entity level | Entire model |
| --- | --- |

| **Name** | **Expression** | **Unit** | **Description** |
| --- | --- | --- | --- |
| Tr | if(abs(z)<=100[um], Trw, Trb) |  | Lambert-Beer law |
| Ab | mu/(pi*SDx*SDy) | 1/m³ |  |
| Gauss_space | exp(-(x^2/(2*SDx^2) + y^2/(2*SDy^2))) |  | gaussian distribution |
| Pw | Q0*(1 - Rc)*(0.5)*Gauss_space*Tr*Ab*gloss | W/m³ | laser heat |
| mu | if(abs(z)<=100[um], uaw, uab) | 1/m | absorption coefficient |
| SDy | 1.02[mm] | m | standard deviation (distance from mean point) (spot radius) |
| SDx | 1.65[mm] | m |  |
| gloss | if(abs(z)<=100[um], rho_lw^2/(rho_lw + abs(z))^2, rho_lb^2/(rho_lb + abs(z))^2) |  |  |
| rho_lw | sqrt((nw/NA)^2 - 1)*0.1*10^(-3)[m] | m |  |
| rho_lb | sqrt(((nb/nw)/NA)^2 - 1)*0.1*10^(-3)[m] | m |  |
| Trw | exp(-uaw*abs(z)) |  |  |
| b | sqrt(a^2 - 1) |  |  |
| a | (usb + uab)/usb |  |  |
| Trb | 2/((sqrt(1 - 1/(a^2)) + 1)*exp(usb*b*abs(z)) + (sqrt(1 - 1/(a^2)) - 1)*exp(usb*b*abs(z))) |  |  |
| usb | 10[cm^ - 1] | 1/m |  |
| uaw | 95.86[1/cm] | 1/m |  |
| uab | 97.68[1/cm] | 1/m |  |

- 1. Functions
     1. Rectangle 1

| Function name | rect1 |
| --- | --- |
| Function type | Rectangle |


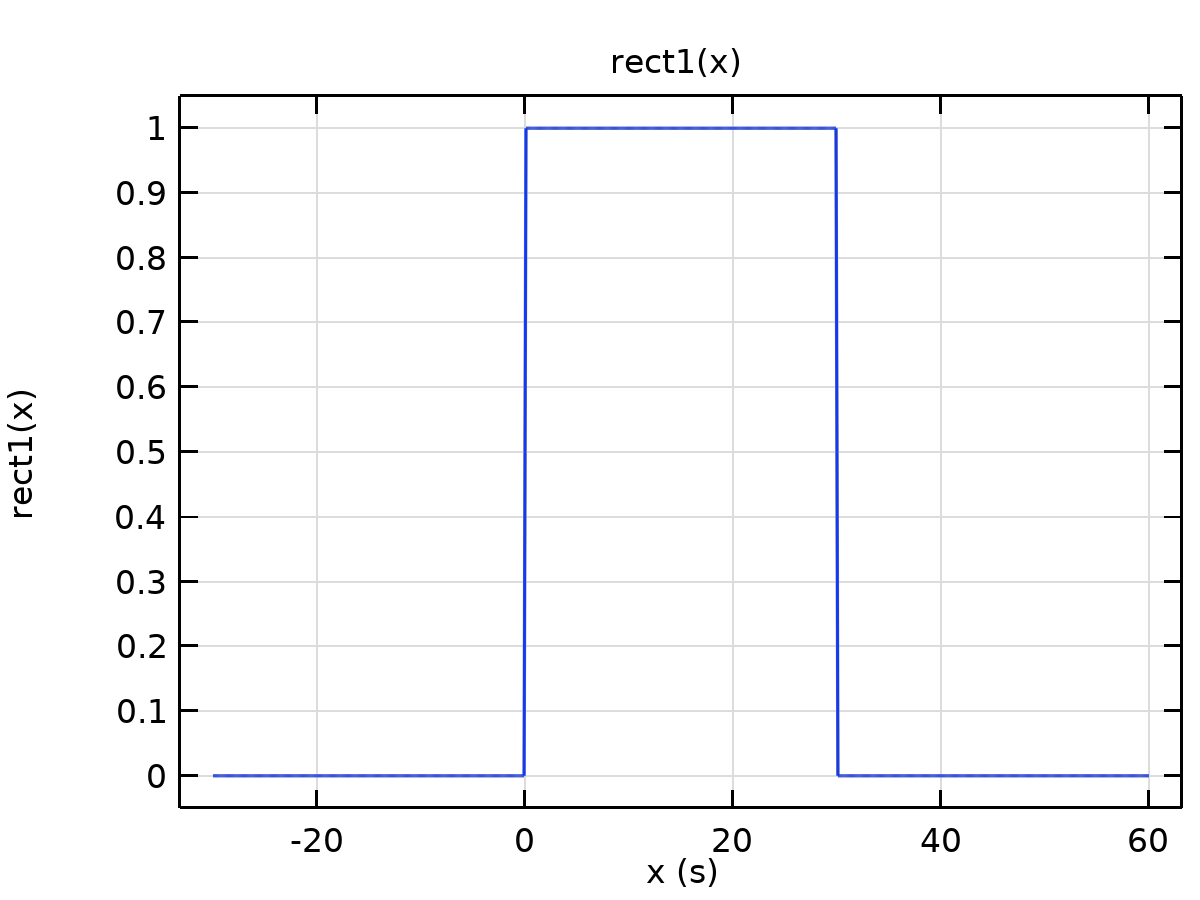


Rectangle 1

- - 1. Analytic 4

| Function name | in1 |
| --- | --- |
| Function type | Analytic |


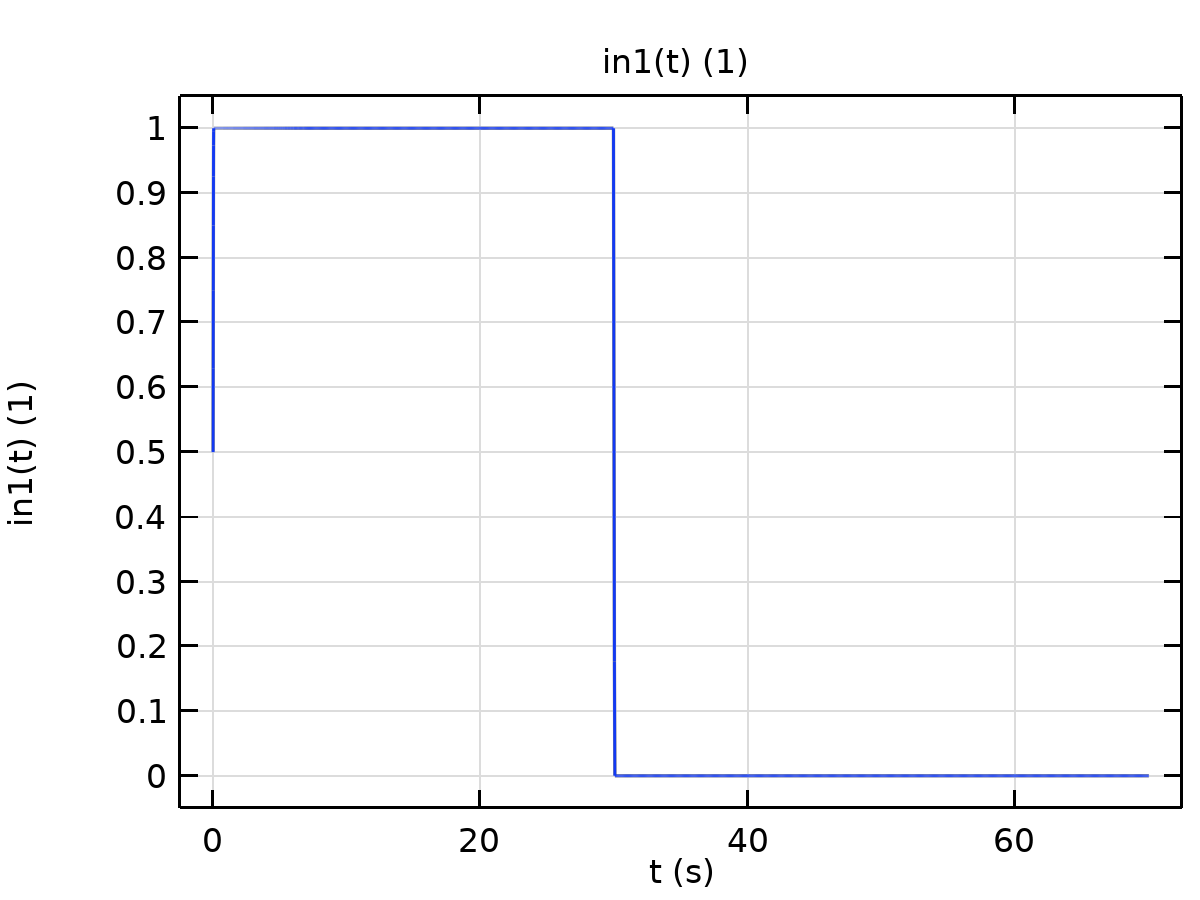


Analytic 4

1. Component 1
   1. Definitions
      1. Coordinate Systems

#### Boundary System 1

| Coordinate system type | Boundary system |
| --- | --- |
| Tag | sys1 |

Coordinate names

| **First** | **Second** | **Third** |
| --- | --- | --- |
| t1 | t2 | n |

- 1. Geometry 1


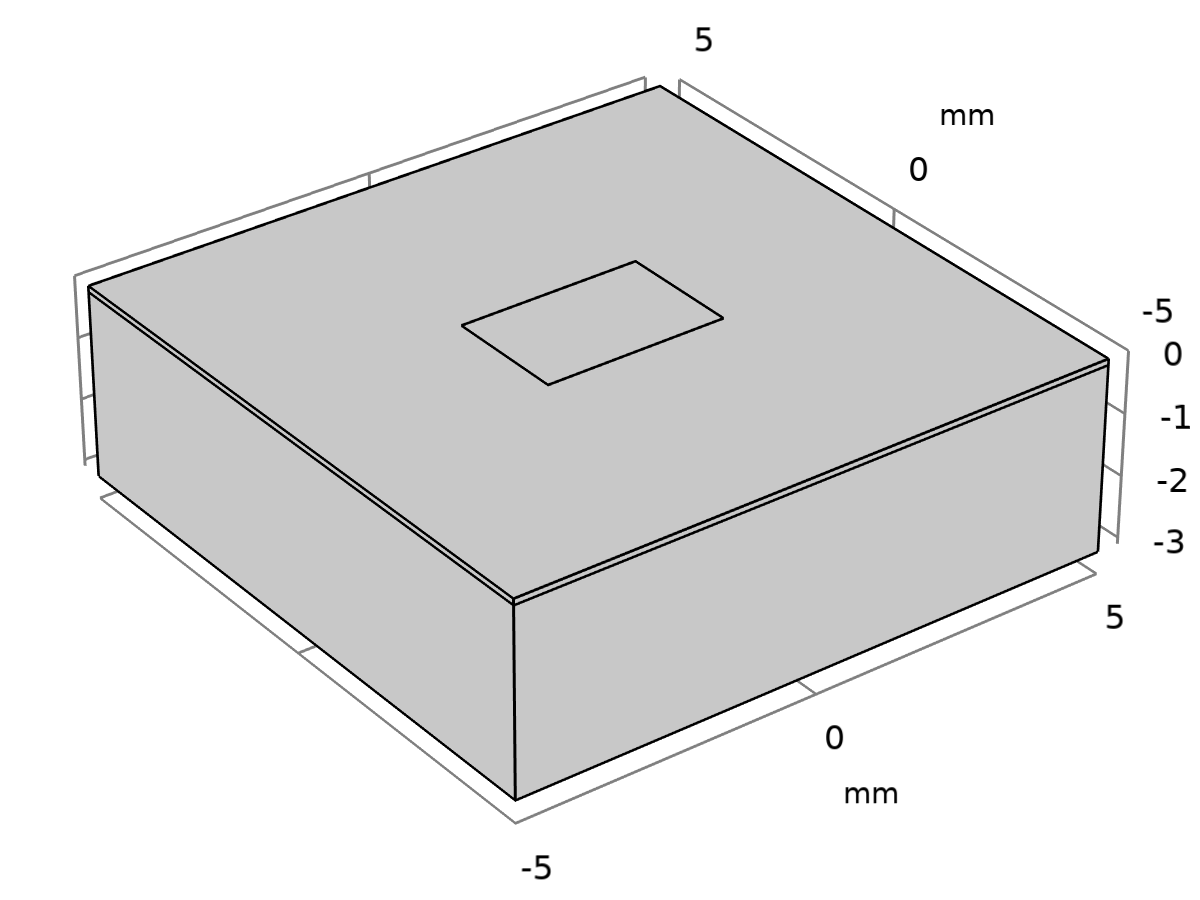


Geometry 1

Units

| Length unit | mm |
| --- | --- |
| Angular unit | deg |

- 1. Materials
     1. mice brain


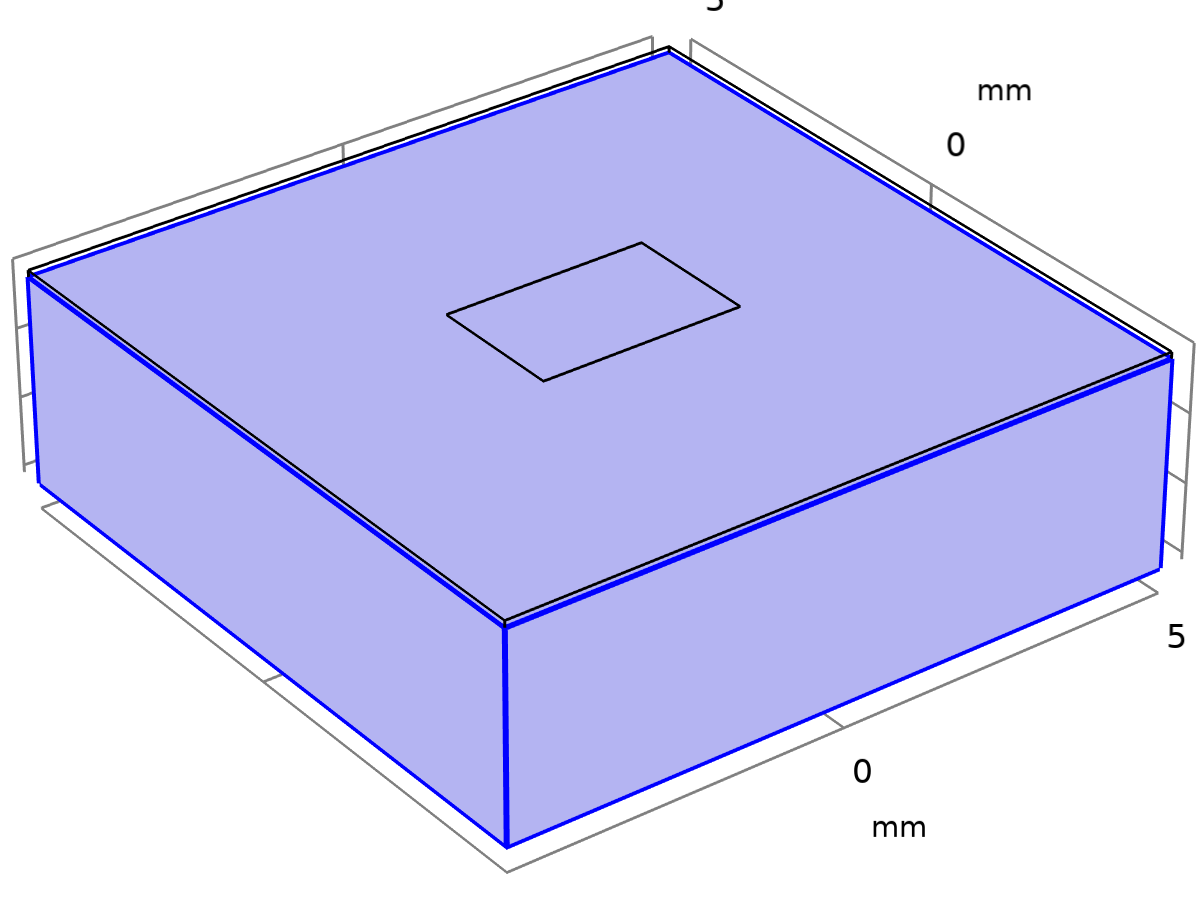


mice brain

Selection

| Geometric entity level | Domain |
| --- | --- |
| Selection | Geometry geom1: Dimension 3: Domain 1 |

- - 1. Water


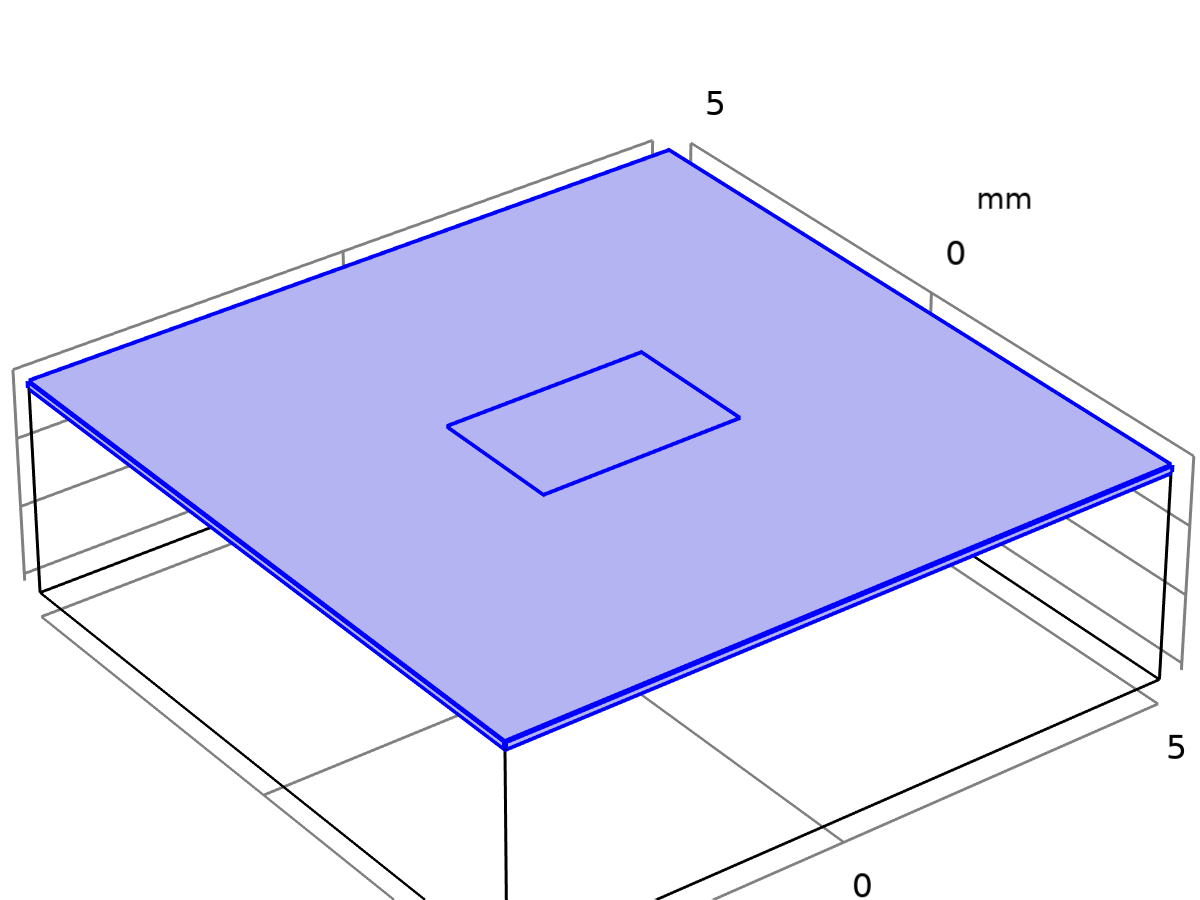


Water

Selection

| Geometric entity level | Domain |
| --- | --- |
| Selection | Geometry geom1: Dimension 3: Domain 2 |

- 1. Bioheat Transfer


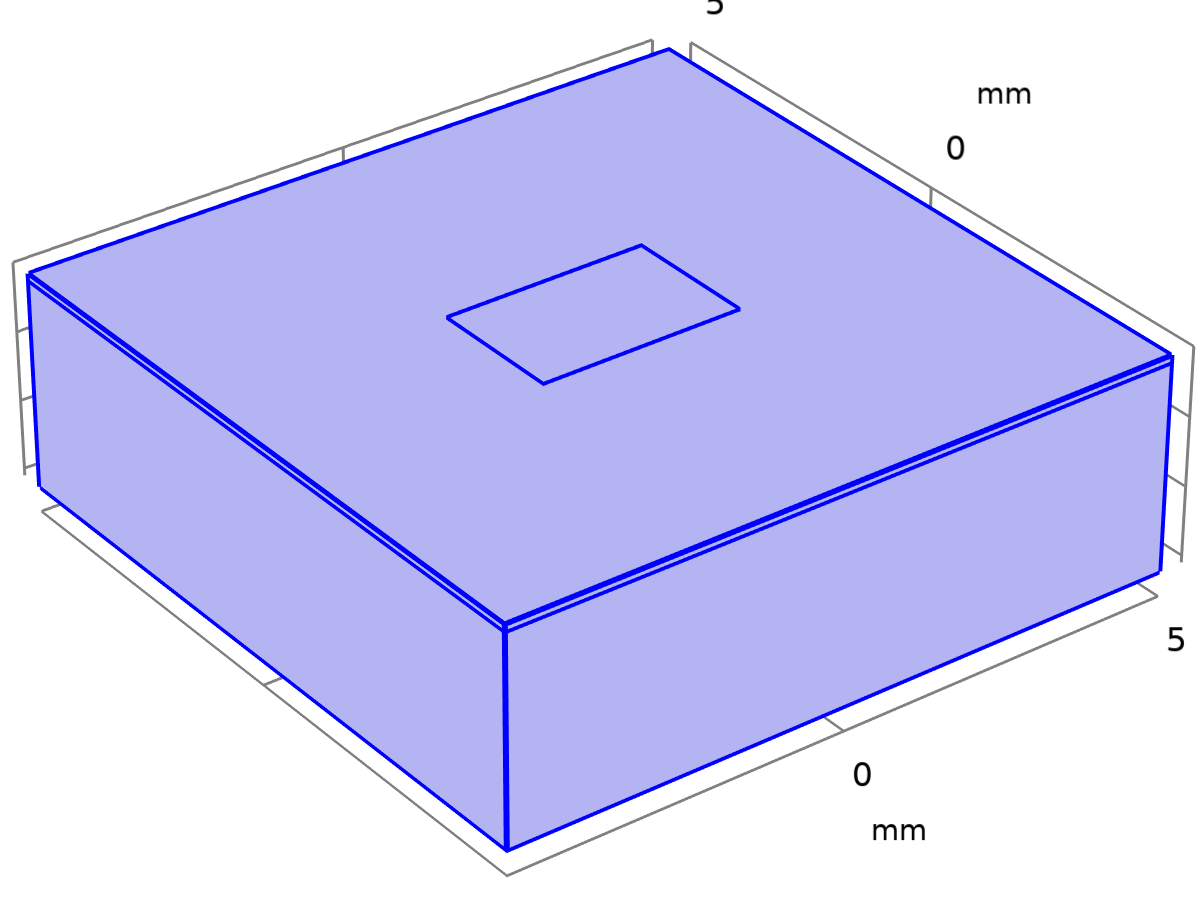


Bioheat Transfer

Equations


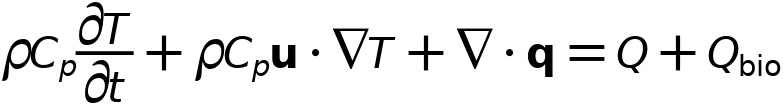


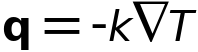


Features

| **Name** | **Level** |
| --- | --- |
| Biological Tissue 1 | Domain |
| Initial Values 1 | Domain |
| Thermal Insulation 1 | Boundary |
| Fluid 1 | Domain |
| Heat Flux 1 | Boundary |
| Heat Source 1 | Domain |

- 1. Events


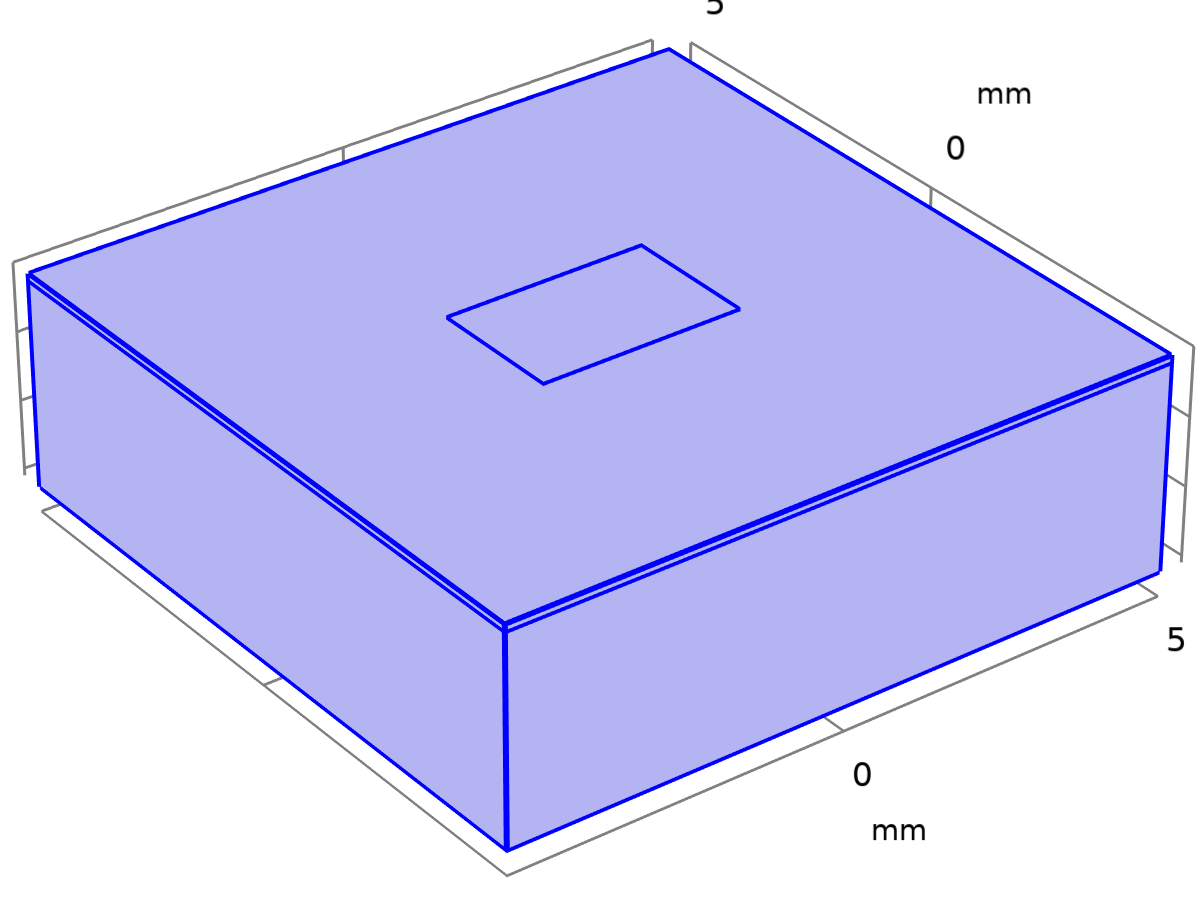


Events

Features

| **Name** | **Level** |
| --- | --- |
| Discrete States 1 |  |
| Explicit Event 1 |  |
| Explicit Event 2 |  |

- 1. Mesh 2


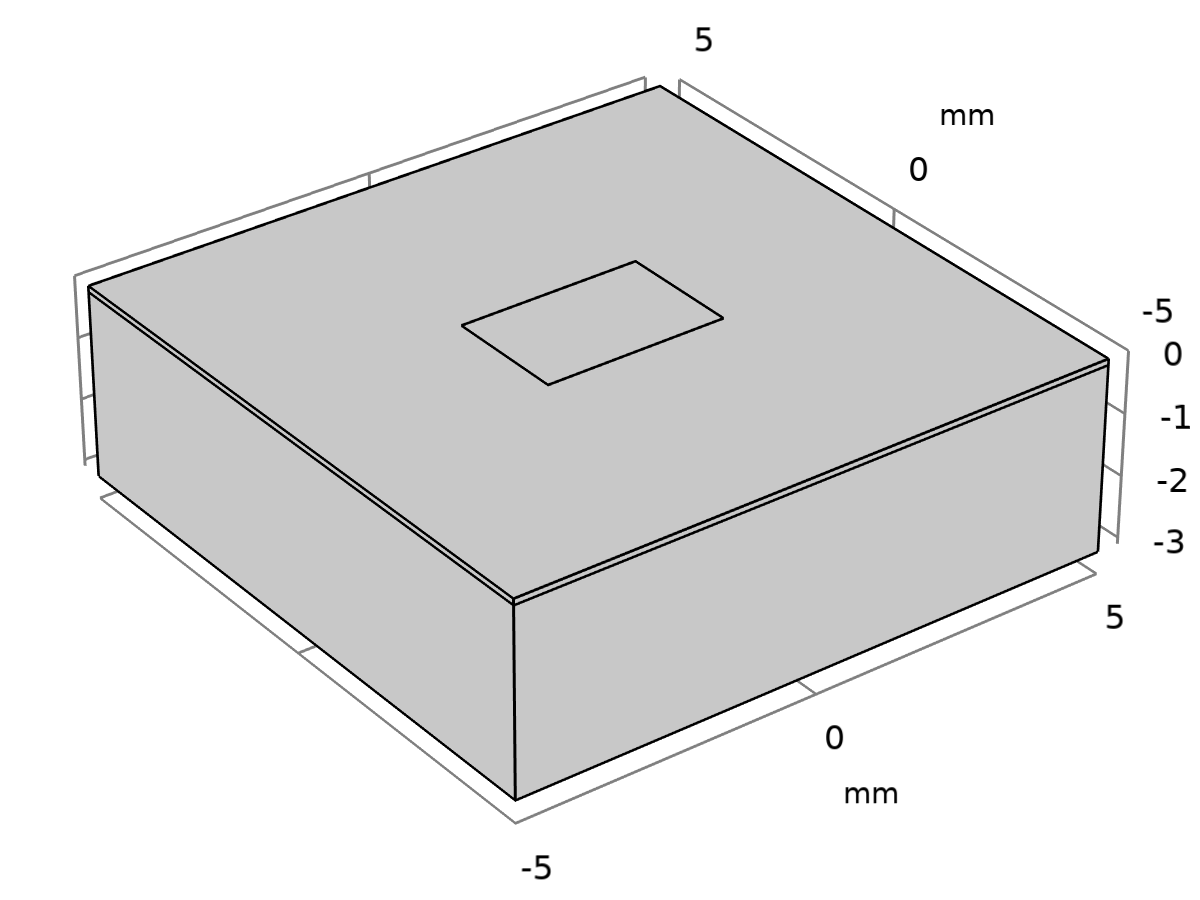


Mesh 2

1. Study 1

Computation information

| Computation time | 57 min 16 s |
| --- | --- |

- 1. Parametric Sweep

| **Parameter name** | **Parameter value list** | **Parameter unit** |
| --- | --- | --- |
| ts | 70,70,70 | s |
| Q0 | 60,70,80 | mW |

Study settings

| **Description** | **Value** |
| --- | --- |
| Sweep type | Specified combinations |
| Parameter name | {ts, Q0} |
| Unit | {s, mW} |

Parameters

| **Parameter name** | **Parameter value list** | **Parameter unit** |
| --- | --- | --- |
| ts (simulation time) | 70,70,70 | s |
| Q0 (laser power) | 60,70,80 | mW |

- 1. Time Dependent

| **Times** | **Unit** |
| --- | --- |
| range(0,0.2, ts) | s |

Study settings

| **Description** | **Value** |
| --- | --- |
| Include geometric nonlinearity | Off |

Study settings

| **Description** | **Value** |
| --- | --- |
| Output times | {0, 0.2, 0.4, 0.6, 0.8, 1, 1.2, 1.4, 1.6, 1.8, 2, 2.2, 2.4, 2.6, 2.8, 3, 3.2, 3.4, 3.6, 3.8, 4, 4.2, 4.4, 4.6, 4.8, 5, 5.2, 5.4, 5.6, 5.8, 6, 6.2, 6.4, 6.6, 6.8, 7, 7.2, 7.4, 7.6, 7.8, 8, 8.2, 8.4, 8.6, 8.8, 9, 9.2, 9.4, 9.6, 9.8, 10, 10.2, 10.4, 10.6, 10.8, 11, 11.2, 11.4, 11.6, 11.8, 12, 12.2, 12.4, 12.6, 12.8, 13, 13.2, 13.4, 13.6, 13.8, 14, 14.2, 14.4, 14.6, 14.8, 15, 15.2, 15.4, 15.6, 15.8, 16, 16.2, 16.4, 16.6, 16.8, 17, 17.2, 17.4, 17.6, 17.8, 18, 18.2, 18.4, 18.6, 18.8, 19, 19.2, 19.4, 19.6, 19.8, 20, 20.2, 20.4, 20.6, 20.8, 21, 21.2, 21.4, 21.6, 21.8, 22, 22.2, 22.4, 22.6, 22.8, 23, 23.2, 23.4, 23.6, 23.8, 24, 24.2, 24.4, 24.6, 24.8, 25, 25.2, 25.4, 25.6, 25.8, 26, 26.2, 26.4, 26.6, 26.8, 27, 27.2, 27.4, 27.6, 27.8, 28, 28.2, 28.4, 28.6, 28.8, 29, 29.2, 29.4, 29.6, 29.8, 30, 30.2, 30.4, 30.6, 30.8, 31, 31.2, 31.4, 31.6, 31.8, 32, 32.2, 32.4, 32.6, 32.8, 33, 33.2, 33.4, 33.6, 33.8, 34, 34.2, 34.4, 34.6, 34.8, 35, 35.2, 35.4, 35.6, 35.8, 36, 36.2, 36.4, 36.6, 36.8, 37, 37.2, 37.4, 37.6, 37.8, 38, 38.2, 38.4, 38.6, 38.8, 39, 39.2, 39.4, 39.6, 39.8, 40, 40.2, 40.4, 40.6, 40.8, 41, 41.2, 41.4, 41.6, 41.8, 42, 42.2, 42.4, 42.6, 42.8, 43, 43.2, 43.4, 43.6, 43.8, 44, 44.2, 44.4, 44.6, 44.8, 45, 45.2, 45.4, 45.6, 45.8, 46, 46.2, 46.4, 46.6, 46.8, 47, 47.2, 47.4, 47.6, 47.8, 48, 48.2, 48.4, 48.6, 48.8, 49, 49.2, 49.4, 49.6, 49.8, 50, 50.2, 50.4, 50.6, 50.8, 51, 51.2, 51.4, 51.6, 51.8, 52, 52.2, 52.4, 52.6, 52.8, 53, 53.2, 53.4, 53.6, 53.8, 54, 54.2, 54.4, 54.6, 54.8, 55, 55.2, 55.4, 55.6, 55.8, 56, 56.2, 56.4, 56.6, 56.8, 57, 57.2, 57.4, 57.6, 57.8, 58, 58.2, 58.4, 58.6, 58.8, 59, 59.2, 59.4, 59.6, 59.8, 60, 60.2, 60.4, 60.6, 60.8, 61, 61.2, 61.4, 61.6, 61.8, 62, 62.2, 62.4, 62.6, 62.8, 63, 63.2, 63.4, 63.6, 63.8, 64, 64.2, 64.4, 64.6, 64.8, 65, 65.2, 65.4, 65.6, 65.8, 66, 66.2, 66.4, 66.6, 66.8, 67, 67.2, 67.4, 67.6, 67.8, 68, 68.2, 68.4, 68.6, 68.8, 69, 69.2, 69.4, 69.6, 69.8, 70} |

Physics and variables selection

| **Physics interface** | **Solve for** | **Equation form** |
| --- | --- | --- |
| Bioheat Transfer (ht) | On | Automatic (Time dependent) |
| Events (ev) | On | Automatic (Time dependent) |

Mesh selection

| **Component** | **Mesh** |
| --- | --- |
| Component 1 | Mesh 2 |
